# Strategies for overcoming multiple barriers of oral administration of protein and peptide therapeutics

**DOI:** 10.1016/j.mtbio.2026.102763

**Published:** 2026-01-07

**Authors:** Xiaofan Wang, Keke Wang, Yitan Fang, Youxi Zhang, Lixin Yi, Xiaohong Li, Qinfu Zhao, Xu Zhu, Shuang Cai, Long Wan

**Affiliations:** a Department of Pharmacy, The First Hospital of China Medical University, 155 Nanjing North Street, Shenyang, 110001, Liaoning, China; b Department of Pharmacy, The Fourth Affiliated Hospital of China Medical University, No. 4 Chongshan East Road, Shenyang, 110032, Liaoning, China; c School of Pharmacy, China Medical University, No. 77 Puhe Road, Shenyang, 110122, Liaoning, China; d Department of Cardiac Surgery, The First Hospital of China Medical University, 155 Nanjing North Street, Shenyang, 110001, Liaoning, China; e Department of Pharmaceutics, School of Pharmacy, Shenyang Pharmaceutical University, 103 Wenhua Road, Shenyang, 110016, Liaoning, China

**Keywords:** Oral administration, Protein and peptide therapeutics, Multiple barriers, Strategies

## Abstract

Oral delivery of protein and peptide therapeutics (PPs) offers a non-invasive, patient-friendly alternative to parenteral administration, yet faces multiple formidable barriers due to the complex gastrointestinal (GI) environment. This review provides an overview of the latest advances of strategies developed to overcome the four main GI barriers: the harsh pH environment, enzymatic degradation, the mucus layer, and the intestinal epithelial barrier. We critically evaluate various approaches including chemical modifications, permeation enhancers, encapsulation techniques, and novel delivery systems like microneedles and nanoparticle-based carriers, each designed to protect PPs and enhance their bioavailability. A comparative analysis of the advantages and limitations of these strategies is presented, along with a discussion on how to integrate them synergistically for the effective overcoming of multiple GI tract barriers. Furthermore, we examine the current challenges associated with oral PPs drug delivery systems and explore future directions aimed at achieving successful therapeutic outcomes.

## Graphical abstract

**Figure:**
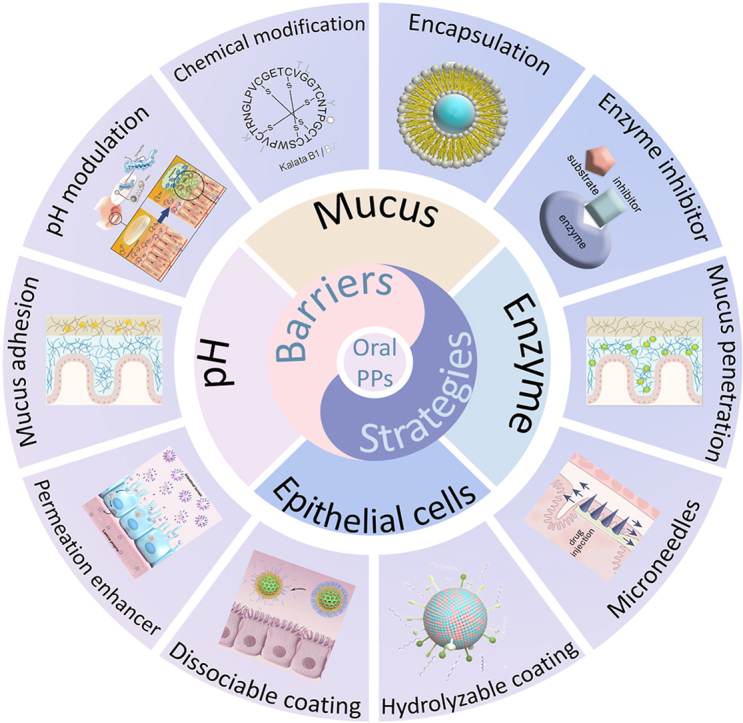

## 1 Introduction

Insulin underwent a 61-year journey from discovery to clinical application, however, its widespread clinical use has driven rapid advancements in protein- and peptide-based drugs [1]. Compared to conventional small-molecule drugs, protein- and peptide-based medications offer a host of advantages, including well-defined mechanisms of action, exceptional selectivity, heightened therapeutic efficacy, enhanced safety profiles, and minimal side effects [2]. Moreover, with the continuous progress of gene editing and microbial fermentation technology, the research, development, and production of PPs are expected to become more efficient, precise, and cost-effective [3]. To date, the majority of PPs are administered through parenteral routes due to the multiple barriers that hinder PPs absorption in the GI tract [4]. The need for continuous subcutaneous insulin injections can cause considerable discomfort and pain for patients, often resulting in adverse effects such as subcutaneous nodules, muscle atrophy, lipohypertrophy, lipoatrophy, scars and infections [5]. This situation has resulted in a decline in patients' medication adherence, subsequently leading to treatment failures. Oral delivery stands as the most prevalent non-invasive drug administration method, offering advantages like convenience, cost-effectiveness, and high patient adherence, making it the preferred route for most medications [6]. Moreover, when it comes to protein drugs, oral delivery can replicate the natural secretion process of proteins, minimizing adverse effects regarding to subcutaneous injections [7]. Given the growing significance of protein-based medications, the development of a practical, reliable, and painless non-invasive oral delivery system is crucial. However, there are numerous formidable barriers within the GI tract that must be overcome for successful oral absorption of PPs.

**Scheme 1:**
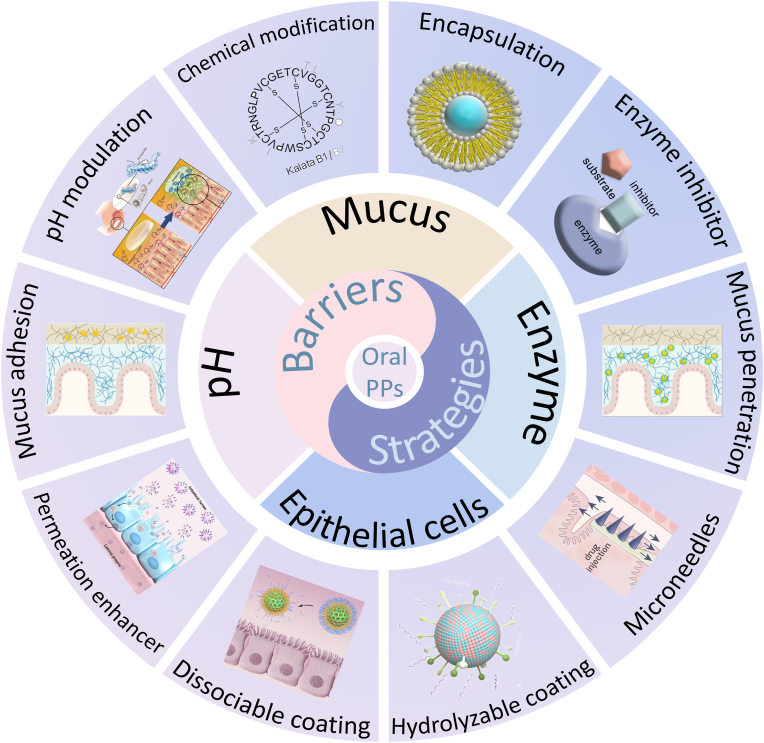
The multiple barriers encountered by oral PPs and the strategies to overcome them.

The harsh GI environment poses a series of challenges to the oral absorption of PPs, such as extreme pH environment in the stomach, proteases and peptidases everywhere, thiol/disulfide exchange reactions, being cleared by the mucus, poor membrane permeability [8]. Despite these formidable challenges, numerous viable methods have been proposed to overcome these obstacles. Several reviews on the research progress of orally administered PPs have been published. However, a comprehensive and systematic review specifically focusing on the GI barriers encountered during the oral administration of PPs and targeted solutions for each barrier is still lacking. This review aims to focus on the multiple barriers of oral delivery of PPs, innovatively summarizing the strategies employed to address each barrier, and providing a theoretical basis for developing effective oral delivery systems for PPs. To help researchers better understand the multiple barriers of oral delivery of PPs and their solutions, this review intends to provide a systematic overview of the research progress on the various strategies employed to effectively overcome these barriers in the oral delivery of PPs and analysis of the advantages and disadvantages of these strategies. Initially, the biochemical, mucus, and epithelial barriers that hinder the oral absorption of PPs are systematically discussed. Subsequently, it delves into the strategies developed to overcome these obstacles, including chemical modifications, encapsulation techniques, pH modulation, enzyme inhibitors, and advanced nanoparticle-based delivery systems. The integration of these strategies into a multi-functional delivery system that promises to improve the bioavailability and therapeutic efficacy of orally administered PPs is further discussed. Finally, the prospects for future research and the challenges that remain in developing these advanced delivery systems are discussed. This review provides a solid foundation for future research directions in the field of orally administered PPs, offering insights that can guide the design of more effective and reliable oral delivery systems for PPs (Scheme 1).

## 2 Barriers for oral administration of protein and peptide therapeutics

### 2.1 Biochemical barrier

**Fig. 1:**
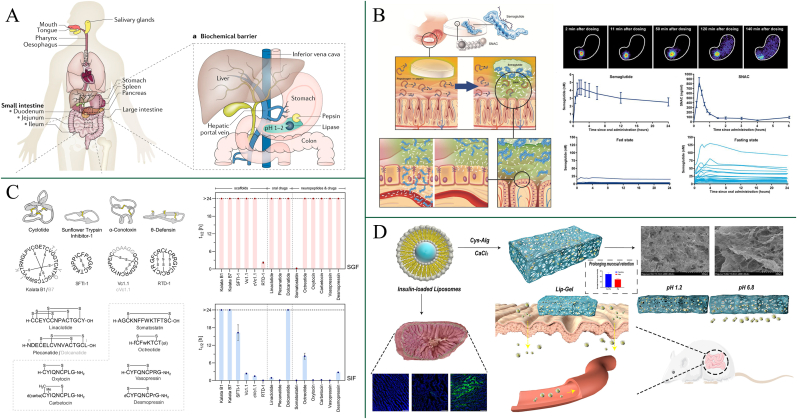
(A) Schematic diagram of the physiological barriers to oral protein and peptide delivery [9]. Copyright 2019 Nature. (B) Buffering effect of SNAC neutralizing the low pH of gastric fluid to protect the co-formulated semaglutide [10]. Copyright 2018 Science. (C) Stability of cyclic peptides in SIF and SGF [11]. Copyright 2022 American Chemical Society. (D) Liposome-in-alginate hydrogels effectively protect encapsulated arginine-insulin complexes from the low pH in the stomach [12]. Copyright 2023 Elsevier.

From the outset, the GI tract's physiology is inherently designed to break down large molecules into their elemental constituents, facilitating nutrient absorption for use throughout the body (Fig. 1A). Therefore, it is not unexpected that the oral bioavailability of whole peptides or proteins is typically less than 1 %. In some cases, it can be even lower than 0.1 % [9]. The initial challenge in the oral administration of PPs is the biochemical barrier, which is primarily defined by the extreme pH environment, the presence of various proteolytic enzymes and thiol/disulfide exchange reactions.

#### 2.1.1 pH barrier

The highly acidic environment in the stomach (pH 1.2–3.0) is one of the first obstacles for PPs. The acidic conditions can cause pH-induced unfolding of protein structures, leading to the exposure of hydrophobic regions that can trigger aggregation or precipitation. Additionally, these acidic conditions can promote oxidative reactions, deamidation, and hydrolysis, all of which compromise the stability and bioactivity of the proteins [13]. Zhang et al. [14] systematically evaluated the stability of insulin under simulated gastric conditions (HCl/NaCl, pH ∼1–2) and demonstrated that intramolecular disulfide bonds of insulin are readily cleaved in gastric juice. This acid-induced disruption of disulfide linkages resulted in rapid structural denaturation and loss of bioactivity before insulin could reach the small intestine, thereby severely limiting its systemic exposure and oral bioavailability. As PPs progress into the intestines, they encounter a more alkaline environment (pH 6.5–8.0). These alkaline conditions can disrupt protein stability by promoting deprotonation of amino acid side chains, which may alter intramolecular interactions and lead to conformational changes or partial unfolding. Benet et al. [15] investigated the solution stability of the GLP-1 receptor agonist exenatide over a gastrointestinal-relevant pH range (4.5–8.5). Exenatide remained relatively stable at pH 4.5. In contrast, exposure to near-neutral conditions (pH 5.5–6.5) markedly accelerated oxidative degradation of exenatide. At higher pH values (7.5–8.5), pronounced deamidation and aggregation were observed, leading to a substantial loss of intact peptide over time. Such structural alterations ultimately compromise the stability and therapeutic efficacy of the orally administered PPs. These drastic pH fluctuations make the GI environment a highly unfavorable barrier for the oral administration of PPs.

#### 2.1.2 Enzyme barrier

The GI tract contains a wide variety of proteolytic enzymes, such as pepsin in the stomach and trypsin, chymotrypsin, elastase, and carboxypeptidases A and B in the small intestine. These enzymes are essential for the digestion of proteins, but they also pose a significant threat to the stability of oral therapeutic peptides and proteins. Proteolytic enzymes rapidly degrade PPs into smaller fragments, often rendering them inactive before they can be absorbed. Zhang et al. examined free insulin in simulated gastric fluid containing pepsin and reported that only 34.07 % of intact insulin remained after 10 min, 1.17 % after 30 min, and no detectable insulin peak was observed after 60 min, highlighting the rapid enzymatic digestion of insulin in the stomach [16]. Similarly, Mudassir et al. investigated the stability of native insulin in simulated intestinal fluid containing trypsin and chymotrypsin. Results showed that free insulin was almost completely degraded within the first 15 min of incubation, indicating that unprotected insulin is extremely susceptible to luminal proteolysis under intestinal conditions [17]. In a complementary study, Liu et al. [18] employed an in vitro pancreatin assay to mimic the intestinal enzyme milieu and characterize the degradation of the oral GLP-1 receptor agonist MEDI7219. They identified 13 peptide metabolites corresponding to eight distinct cleavage sites, which were consistent with the specificities of trypsin-, pepsin-, and elastase-like proteases. These results indicate that even engineered GLP-1 analogues are susceptible to extensive proteolysis in gastrointestinal enzyme-rich environments. Collectively, these findings demonstrate that GI enzymes constitute a particularly formidable barrier during the oral administration of PPs.

#### 2.1.3 Thiol/disulfide exchange reactions barrier

Besides pH fluctuations and enzymatic degradation, therapeutic peptides and proteins that contain thiol and/or disulfide substructures are particularly vulnerable to thiol/disulfide exchange reactions in the GI tract [8]. Under physiological conditions, reduced glutathione and other thiol-containing species are abundant in the intestinal lumen. These reducing agents can disrupt the native disulfide bonds within protein structures, thereby destabilizing their folded conformation. At the same time, aberrant thiol/disulfide exchange can induce the formation of non-native disulfide linkages or conjugates with other luminal molecules, leading to irreversible structural modifications. Such alterations often abolish the biological activity of PPs and may even accelerate their clearance or aggregation. Ijaz et al. [19] incubated the cyclic somatostatin analogue lanreotide with reduced glutathione and thiol-enriched casein peptones under gastrointestinal-relevant conditions. They observed a rapid loss of native lanreotide accompanied by the formation of mixed disulfide conjugates. Likewise, Schmitz and co-workers [20] used LC–MS-based analysis to characterize the thiol–disulfide chemistry of desmopressin. Under physiological concentrations of glutathione, desmopressin bearing a single disulfide bond rapidly formed multiple mixed disulfide conjugates, leading to a marked depletion of the intact peptide. These studies demonstrate that endogenous thiols such as glutathione, together with thiol-containing dietary proteins and mucus glycoproteins, can drive extensive thiol/disulfide exchange of disulfide-rich PPs in the GI lumen, generating inactive conjugates and markedly reducing the fraction of bioactive PPs available for absorption. Consequently, thiol/disulfide exchange reactions represent an additional biochemical challenge that severely compromises the stability and therapeutic efficacy of orally administered PPs.

### 2.2 Mucus barrier

If PPs manage to evade degradation by proteolytic enzymes, they have to subsequently traverse the intestinal mucus, which lines on the entire intestinal epithelium [9]. Mucus is comprised of mucin, enzymes, electrolytes and water, serving as a lubricant and a protective shield for intestines. Mucin is the main component of mucus, secreted by goblet cells and submucosal glands. Mucin molecules entangle and crosslink adhesively and reversibly to form a dynamic viscoelastic gel with shear-thinning properties. This is the main reason why the mucus layer is viscoelastic. Intact proteins or other foreign substances are easily to be trapped in mucus with different noncovalent interactions, such as hydrogen bonding, electrostatic, size exclusion and hydrophobic interactions [21]. Furthermore, the outer layer of mucus is loose, flowing and periodically cleared, so that the trapped proteins and foreign substances will be removed quickly [22]. These characteristics make the mucus layer a very difficult barrier to deal with during the oral administration of PPs.

### 2.3 Epithelial barrier

If PPs successfully overcome the biochemical barriers within the GI tract and traverse the mucus layer, they reach to the final and most challenging GI barrier of oral protein delivery – the epithelial cell barrier. Typically, PPs can cross epithelial monolayer through two main pathways: the paracellular route (moving between cells) and the transcellular route (passing through cells) [23]. At first glance, the paracellular route appears to be more compatible with the transport of peptide or protein drugs, for the mechanism is simpler and more straightforward. Simply opening the tight junctions between cells allows peptides or protein drugs to pass through. However, tight junctions only occupy <1 % of the total mucosal surface and the gap is about 6 Å, which is too small for proteins to pass [24]. For the transcellular route, PPs are unable to effectively partition into the lipid bilayers of epithelial cells as a result of their hydrophilic properties, thereby significantly hindering their cellular uptake [25]. Even if the endocytosis of proteins is achieved, the proteins still have to evade expulsion back into the intestinal lumen by efflux pumps within enterocytes and avoid being degraded by intracellular proteases [26]. After a successful transport of endocytic proteins within the intestinal cells, sufficient basolateral exocytosis is necessary to complete the efficient transepithelial transport of oral peptide and protein drugs [27].

## 3 Strategies for overcoming barriers of orally administered protein and peptide therapeutics

### 3.1 Strategies for overcoming biochemical barrier

The GI tract presents a series of biochemical barriers that challenge the effective oral delivery of PPs. These barriers include extreme pH conditions, enzymatic degradation, and thiol/disulfide exchange reactions. To ensure the stability of PPs, strategies must be developed to protect them from these harsh environments. Biochemical barriers are particularly difficult to overcome due to the complexity and variability of the GI environment, where pH fluctuates, digestive enzymes are everywhere and thiol/disulfide exchange reactions occur. To address these challenges, various strategies have been developed to enhance the stability of orally administered PPs. These strategies include chemical modifications to improve protein stability, the use of pH-modulating agents to protect PPs from extreme acidity, and encapsulation techniques to shield the proteins from enzymatic degradation and thiol/disulfide exchange reaction. In this section, pH modulation and chemical modification are analyzed within both extreme pH barrier and enzyme barrier. pH modulation is an environment-centric approach that adjusts local microenvironmental pH to stabilize PPs or to attenuate proteolytic activity. Chemical modification is molecule-centric, tailoring the intrinsic properties of PPs (e.g., steric shielding, terminal/sequence editing, lipidation) to resist acid/base-induced unfolding and enzymatic degradation. The differential operation of each strategy under the extreme pH barrier (Section 3.1.1) and the enzyme barrier (Section 3.1.2) is delineated below, with emphasis on their distinct mechanistic pathways.

#### 3.1.1 Strategies for overcoming extreme pH environment

To function, PPs have to maintain stably folded into their tertiary conformation. When the environment pH is significantly different from the protein's isoelectric point, it makes the protein highly charged resulting in an increase of electrostatic repulsions. This alteration triggers the unfolding and aggregation of the protein, ultimately leading to its inactivation [28]. The pH varies significantly throughout the GI tract. Gastric fluids are highly acidic with a pH ranging from 1.5 to 3.5. Within the duodenum, the pH increases to around 5 to 6 due to the neutralization of carbonate and bile juices. In the distal jejunum and ileum, the pH further rises to approximately 7–8. In the colon, the pH can even exceed 8. The complex pH environment within the GI tract makes oral administration of PPs exceedingly challenging. The extreme pHs lead to unfolding and protonation of proteins, resulting in stability decreases even inactivation [29]. And the improved ionic strength of GI tract would enhance the aggregation of PPs because of strengthened hydrophobic interactions [30]. Despite the extreme GI pH conditions posing a significant threat to orally administered PPs, up to now, there are still three effective strategies to overcome the pH barrier: (1) pH modulation; (2) chemical modification; (3) encapsulation.

##### 3.1.1.1 pH modulation

The first strategy to preserve PPs in the extreme pH environment of GI tract is pH modulation. This strategy involves adjusting the local pH microenvironment around the PPs to preserve their structures and efficacy. By modulating the pH, PPs can remain stable in the highly acidic environment of the stomach, and maintain their activity in the intestines. This is typically achieved using buffering agents or other pH modulators, which create a transient protective microenvironment around the dosage form and thereby help maintain drug activity and stability during transit through the GI lumen. In clinical practice, a pH buffer is commonly employed to ameliorate the highly acidic gastric environment [31], enabling PPs to bypass the acidic barrier.

pH buffers offer several advantages for GI environment modulation. They are generally composed of well-characterized, pharmaceutically acceptable excipients, which simplifies safety evaluation and regulatory approval. Buffers can be readily incorporated into conventional oral dosage forms and can be combined with other strategies such as permeation enhancers or enzyme inhibitors without requiring complex manufacturing processes. However, the use of pH buffers for GI modulation also has limitations. First, the buffer capacity is constrained by the amount of excipient that can be incorporated into a single dosage form and may be insufficient to neutralize the large and variable volume of gastric fluid. As a result, the achieved pH shift is often modest, localized, and short-lived, and can be strongly influenced by inter- and intra-individual differences in gastric volume, basal pH, and gastric emptying. Second, high buffer loads may cause local irritation, discomfort, or osmotic effects and can potentially alter the dissolution or absorption of concomitantly administered drugs. Third, in the intestine, strong pH buffering may inadvertently interfere with physiological gradients that are important for digestion and absorption, and may not be sufficient to significantly reduce the activity of multiple luminal proteases over extended segments of the gut.

Consequently, compared with direct pH modulation of the stomach, the use of enteric coating on PPs is generally preferred for overcoming gastric degradation. This preference is attributed to the simpler formulation of enteric coatings, which offer a more cost-effective solution for protecting PPs against the stomach's harsh acidic environment. Tarsa Therapeutics has proposed an enteric-coated capsule for the oral delivery of salmon calcitonin (sCT, ORACAL®), which could efficiently bypass the stomach [32]. Once the system reaches the intestine, PPs are released from their enteric coating.

While the intestinal tract's near-neutral pH does not make PPs unfold and deactivate, the abundant luminal proteases exhibit maximal proteolytic activity at this pH. The activity of these proteases is pH-dependent. For example, the enzymatic activities of trypsin and chymotrypsin, exhibit a gradual decrease as the pH is lowered [33]. Modulating the intestinal pH has emerged as an efficacious strategy for preserving PPs within the intestinal environment. Therefore, Tarsa Therapeutics not only employed an enteric-coated capsule to ensure sCT to circumvent gastric passage, but also incorporated citric acid to modulate the intestinal pH microenvironment. pH modulation effectively enhanced the oral bioavailability of sCT. In addition, oral semaglutide (Rybelsus®) exemplifies gastric pH modulation to protect peptides in the stomach [10]. Co-formulation with sodium N-[8-(2-hydroxybenzoyl) amino caprylate] (SNAC) creates a localized neutral microenvironment around the tablet as it erodes. This buffering effect mitigates gastric acidity and consequently enhances the structural stability of semaglutide. In the context of the pH barrier, pH modulation primarily prevents the unfolding and aggregation of PPs by counteracting acid- or base-induced protonation and electrostatic repulsion.

##### 3.1.1.2 Chemical modification

Chemical modification could also protect PPs from the extreme pH conditions of GI tract. Various chemical modification techniques have been employed, such as N/C-terminal modification, increased molecular mass (lipidation, PEGylation), peptide cyclization and D-amino acid or unnatural amino acid substitution [34]. Hunt et al. [35] conjugated insulin to silver sulfide quantum dots (Ag_2_S QDs), creating a novel insulin formulation (QD-INS). This modification enhanced insulin's stability by reducing its solubility in acidic environments, effectively protecting it from deactivation in the stomach. The resulting QD-INS formulation demonstrated improved bioavailability and liver targeting, with a dose-dependent reduction in blood glucose without inducing hypoglycemia or weight gain in animal models. Li et al. [36] synthesized an antimicrobial peptide L163 by computational design that is very vulnerable, but N-terminally acetylated L163 demonstrates enhanced stability under various pH conditions. N-terminal acetylation played a crucial role in maintaining stability across different pH environments, contributing to the peptide's robustness and functional efficacy in diverse biological settings. Coolich et al. [37] conjugated poly (ethylene glycol) (PEG) to the B29-lysine site of insulin, resulting in PEGylated insulin. This PEGylation modification enhanced insulin's structural integrity by forming a hydrated steric shield that stabilized the molecule in acidic environments and contributed to sustained release from pH-responsive hydrogel matrix in the small intestine. The resulting PEGylated insulin demonstrated improved resistance to degradation and pH-induced destabilization, supporting its potential for effective oral delivery.

Moreover, the other chemical modification methods like lipidation [38], peptide cyclization [39] and D-amino acid substitution [40] are also used to improve the stability of PPs in GI tract. PEGylation provides a hydration-mediated steric shell that reduces solvent and proton access to ionizable surface residues, thereby dampening acid/base–driven electrostatic repulsion and suppressing unfolding/aggregation. Cyclization increases backbone rigidity and lowers conformational entropy of unfolding, raising the free-energy barrier to acid- or base-induced denaturation. Lipidation promotes hydrophobic microphase formation, which shields labile motifs from bulk acidic milieu and stabilizes tertiary packing. Chemical modifications preserve native-like folding of PPs under extreme pH conditions, reducing pH-triggered structural destabilization.

##### 3.1.1.3 Encapsulation

Lastly, encapsulation could also preserve the orally administered PPs intact within the GI tract. The encapsulating layer forms a physical barrier around the PPs, shielding them from the harsh gastric fluids. This barrier prevents direct contact with the digestive enzymes and the acidic or alkaline conditions in the GI tract. Most proteins are optimally stable near neutral pH, and deviations to either extreme can cause unfolding, rendering the protein inactive [9]. Therefore, we just need to overcome the extreme acidic environment of stomach. Rasjava et al. [41] developed acetylated inulin nanoparticles (InAc-Ins NPs) to encapsulate insulin, providing a protective barrier against the acidic gastric environment. This encapsulation preserved insulin's secondary and tertiary structures, extending its stability and protecting it from premature degradation. Once in the intestines, the acetylated inulin matrix degraded due to intestinal microbiota, allowing for efficient insulin release. This approach helped maintain insulin's stability and improved its oral bioavailability. Encapsulation strategy protects oral PPs from extreme acidic gastric environment and enhances the oral bioavailability of PPs.

#### 3.1.2 Strategies for overcoming the enzyme barrier

Proteolytic enzymes in the GI tract pose a significant challenge to the stability and efficacy of orally administered PPs. These enzymes are specialized in breaking down proteins into smaller peptides and amino acids, which is a natural process essential for the digestion and absorption of dietary proteins [42]. However, this proteolytic environment is detrimental to therapeutic PPs, as it can lead to their premature degradation, thereby reducing their bioavailability and therapeutic efficacy. Pepsin, operating optimally in the acidic pH of the stomach, cleaves peptide bonds, particularly those adjacent to aromatic amino acids. In the more neutral pH of the small intestine, trypsin and chymotrypsin further degrade proteins and peptides, with trypsin preferentially cleaving at basic amino acid residues and chymotrypsin at aromatic residues. Due to these features, current research has focused on two primary strategies to protect PPs from enzymatic degradation in the GI tract. The first strategy focuses on inhibiting the activity of proteolytic enzymes by incorporating enzyme inhibitors or adjusting the pH. The second strategy seeks to prevent proteolytic enzymes from accessing the active sites of PPs through chemical modification or encapsulation.

##### 3.1.2.1 Addition of enzyme inhibitor

Inhibiting enzyme activity can be achieved through the use of enzyme inhibitors that bind to the active sites of these enzymes and prevent them from interacting with the PPs. A lot of protease inhibitors have been explored, broadly classified into two types: amino acid-based inhibitors (e.g., aprotinin, soybean trypsin inhibitor, U-Omp19) and non-amino acid inhibitors (e.g., camostat mesylate, 4-(2-Aminoethyl) benzenesulfonyl fluoride hydrochloride, ubenimex) [43]. A unique subset of these inhibitors operates through indirect mechanisms, not by binding directly to the proteases but by binding essential ions such as calcium, like ethylene diamine tetraacetic acid (EDTA), thereby indirectly diminishing protease activity [44]. Typically, these inhibitors are integrated into the oral formulation alongside the PPs. Coria et al. [45] devised an effective oral antigen delivery system co-encapsulated with a protease inhibitor to prevent enzymatic degradation of loaded antigen. The recombinant unlipidated outer membrane protein from Brucella spp. (U-Omp19) was introduced as a protease inhibitor in the oral vaccine formulation. Their findings revealed that U-Omp19 inhibits protease activity from murine intestinal brush-border membranes and cysteine proteases from human intestinal epithelial cells (IECs), enhancing the accumulation of co-administered antigen within lysosomal compartments of IECs. Furthermore, they demonstrated that co-administration of U-Omp19 facilitated the transcellular transport of antigen through epithelial cell monolayers both in vitro and *in vivo* without compromising epithelial cell barrier integrity.

However, the utilization of enzyme inhibitors presents several complications. Maintaining an adequately high concentration of the inhibitor to effectively reduce protease activity over an extended duration poses a significant challenge [43]. Furthermore, non-amino acid inhibitors may exhibit toxic properties at elevated concentrations [43]. Additionally, the disruption of natural protein degradation pathways can negatively impact normal digestive processes and nutrient absorption. This may trigger a compensatory response, resulting in an upsurge in protease production with continued use of inhibitors [46]. Above all, although enzyme inhibitors effectively reduce the enzymatic degradation of orally administered PPs, the associated side effects cannot be overlooked.

##### 3.1.2.2 pH modulation

In contrast, when considered under the enzyme barrier, pH modulation does not directly stabilize the structure of PPs, but rather diminishes enzymatic activity by shifting the local pH away from the enzyme's optimal range. Enzymatic activity is highly pH-dependent, with each enzyme displaying optimal function within a specific pH range. For instance, pepsin demonstrates maximum enzymatic activity under highly acidic conditions (pH < 3). When exposed to a less acidic environment, notably at a pH exceeding 3, pepsin's activity diminishes significantly. Conversely, luminal enzymes like trypsin and chymotrypsin are more active in alkaline environments (pH ≥ 6.5). Above all, modulating GI pH can therefore decrease protease activity to protect orally administered PPs. Buckley et al. [10] devised an orally delivered semaglutide tablet coformulated with the buffering agent SNAC (Fig. 1B). Despite SNAC being more commonly recognized as a permeation enhancer, it also functions as a pH modulator. The two pKa values of SNAC (pKa^1^ = 4.5 and pKa^2^ = 8.6) provide it with a local buffering capacity, allowing the deprotonated form of SNAC to neutralize the acidic pH of the stomach. SNAC significantly raises the local microenvironment pH, which in turn reduces the activity of gastric pepsin, thereby protecting semaglutide from degradation. This innovative approach offers a transformative shift from injectable to tablet-based oral therapies for semaglutide, enhancing patient compliance and therapeutic outcomes.

The intestinal enzymes exhibit peak activity in neutral to basic pH conditions. Incorporating citric acid into the tablet formulation induces a localized, temporary reduction in pH, consequently impeding the activity of native intestinal proteases. Notably, the concurrent administration of citric acid with oral salmon calcitonin could certainly increase absorption of salmon calcitonin in beagle models, primarily by attenuating the function of local trypsin-like enzymes [47]. Moreover, itaconic, fumaric, and tartaric acids have also been used to alter the pH of the microenvironment of GI tract to inhibit the protease activity [9]. In summary, modulating the GI pH using buffering agents like SNAC and acids such as citric, itaconic, fumaric, and tartaric acids can effectively reduce protease activity, thereby enhancing the stability and oral bioavailability of PPs.

##### 3.1.2.3 Chemical modification

Within the enzyme barrier, chemical modification protects PPs by shielding or eliminating protease recognition sites, thereby preventing active-site engagement and decreasing the apparent proteolytic rate constant. Preventing PPs from entering the active sites of proteases can be realized by chemical modification of PPs [48]. The necessity of protein degradation hinges on the requirement for proteins to enter the active domain of proteases. For example, prior to the proteolytic degradation of insulin, the protease must effectively bind to the insulin molecule at the right position [49]. Biocon Ltd has modified a single methoxy-trimethylene-ethylene glycol-propionyl unit onto the lysine-β 29-amino group of human insulin via an amide linkage. PEGylated recombinant insulin effectively evades protease recognition, thereby enhancing the stability of the insulin [31]. The covalent attachment of PEG for adaptation to the GI tract environment has emerged as the predominant method for the chemical modification of PPs.

In addition, peptide cyclization an also protect peptides from enzymatic degradation and maintain their stability in the GI tract. Merz et al. [50] used de novo design to generate head-to-tail macrocyclic peptides and showed that these macrocycles displayed markedly improved stability in simulated gastric fluid (pepsin) and simulated intestinal fluid (pancreatin), whereas the corresponding linear analogues were rapidly degraded. Increasing the conformational constraint and backbone rigidity of cyclic peptides is thought to reduce their conformational adaptability within protease active sites, thereby further enhancing their resistance to gastrointestinal enzymatic degradation. Consistent with this mechanistic rationale, Kremsmayr et al. [11] conducted a systematic gut-stability analysis of disulfide-rich peptide scaffolds in gastric and small-intestinal fluids (Fig. 1C). They found that only a small subset of disulfide-rich cyclic scaffolds retained high levels of intact peptide after prolonged incubation in intestinal media, whereas most linear peptides and non-cyclized analogues were rapidly degraded. These results indicate that dense disulfide-based macrocyclization rigidifies the peptide backbone and shields protease-sensitive motifs, thereby conferring pronounced resistance to gastrointestinal enzymatic degradation. Lipidation, deamination [51], prodrugs [4], scaffold grafting [11], N-alkylation [52], acylation and substitution of existing amino acids with new amino acids [11] have also been used as chemical modifications to block contact of PPs with digestive enzymes, improving the stability of oral PPs.

##### 3.1.2.4 Encapsulation

In addition, encapsulation can also achieve effective isolation between PPs and enzymes. Compared with enteric coating, micro/nano-particles don't only protect PPs from pepsin in stomach, but also isolate PPs from enzymes in intestine due to the overall uptake of particles by enterocytes. Wu et al. [12] developed a novel encapsulation strategy to protect encapsulated arginine-insulin complexes from the low pH in the stomach and enzymatic degradation (Fig. 1D). The encapsulation involved using liposomes loaded with arginine-insulin complexes (AINS) incorporated into cysteine-modified alginate hydrogels to form AINS-Lip-Gel. The hydrogel's porous structure could vary with pH, enhancing stability and controlled release of the loaded arginine-insulin. *In vitro* release studies showed that AINS-Lip-Gel released only ∼10 % of insulin at pH 1.2, compared to ∼40 % from AINS-Lip, effectively avoiding initial burst release and providing substantial protection to the encapsulated AINS. Furthermore, *in-vivo* hypoglycemic studies in diabetic mice revealed that AINS-Lip-Gel had a significantly stronger and prolonged hypoglycemic effect, as a result of effective encapsulation that reduced enzymatic degradation of AINS.

Additionally, Abella-López et al. [53] developed a nanoencapsulation platform using an IC-Tagging system to encapsulate the enzyme phenylalanine ammonia lyase (AvPAL) for oral delivery. The nanoencapsulated AvPAL demonstrated enhanced stability in gastrointestinal (GI) environments, resisting acidic conditions and proteolytic degradation. This system effectively maintained AvPAL's enzymatic activity throughout the GI tract, offering a promising strategy for oral enzyme replacement therapies, particularly for treating phenylketonuria (PKU). Menina et al. [54] devised a bacteria-derived invasive moiety modified liposome to encapsulate an antibiotic colistin. The formulated liposomes demonstrated good stability in GI-simulating media, preventing enzymatic degradation of colistin efficiently. This system successfully enhanced the stability of colistin against GI proteases and improved its intracellular delivery, offering a promising strategy for oral delivery of PPs. Moreover, other nanoparticles (NPs) like liposomes [55], nanoemulsions [56], polymeric NPs [13], lipid NPs [8] and inorganic NPs [57] have also been extensively explored for oral macromolecular drug delivery. These findings highlight the potential of encapsulation in improving the oral bioavailability of PPs by protecting them from enzymatic degradation.

#### 3.1.3 Strategies for overcoming thiol/disulfide exchange reactions

Therapeutic PPs with thiol and/or disulfide moieties exhibit susceptibility to thiol/disulfide exchange reactions within the GI tract [51]. This propensity often leads to the generation of inactive conjugates. Such reactions are not limited to interactions with endogenous thiol compounds, including glutathione and cysteine-rich domains of mucus glycoproteins, but also involve dietary proteins. A notable example is Desmopressin, which form three distinct disulfide-linked conjugates with glutathione under physiological conditions [19]. Protection against thiol/disulfide exchange reactions for PPs is also provided by physical isolation. Zhou et al. [58] developed PEG-C13-ME–based nanoparticles to encapsulate insulin, providing a physical barrier against the highly hydrophilic thiol-containing molecules such as glutathione. Owing to the hydrophobic inner core and PEGylated surface shielding, glutathione was unable to access the encapsulated insulin, effectively preventing thiol/disulfide exchange reactions and subsequent protein inactivation. Besides, lipid-based nanocarriers [8] and the NPs that can provide shelter for PPs to avoid enzyme attacks previously discussed in the Section 3.1.2 could also protect oral PPs from thiol/disulfide exchange reactions.

### 3.2 Strategies for overcoming mucus barrier

Mucus is designed to protect the gastric and IECs underneath it. Mucus layer only allows small nutrient molecules or degraded amino acids to pass through, which imposes multifaceted impediments to permeation of orally administered PPs into submucosal tissues [59]. Mucus turnover in the intestine, typically ranging from 50 to 270 min, leads to the removal of PPs or particles trapped in the mucus layer, thereby reducing the adhesion and retention time of PPs [60]. Before reaching to the intestinal epithelium, the ongoing secretion and turnover of mucus layers create significant challenges for PPs attempting to infiltrate through the mucus layer.

Mucin, a constituent glycoprotein with molecular diameters spanning 3–10 nm, constitutes the principal functional element of the mucus [61]. The core polypeptide structure of mucin is predominantly composed of serine and threonine residues, which serve as frequent sites of glycosylation. The appended glycan chains, terminally adorned with sulfate and sialic acid moieties, impart a net negative charge to the mucus. Concurrently, the hydrophobic, non-glycosylated domains of mucin, enriched in cysteine residues, facilitate the formation of disulfide bonds. These linkages are instrumental in the organization of mucin fibers into a complex, interwoven meshwork, characterized by a crosslinked and bundled configuration. The resultant mucus network exhibits a heterogeneous pore architecture, with dimensions varying from 20 nm to 1800 nm [62].

**Fig. 2:**
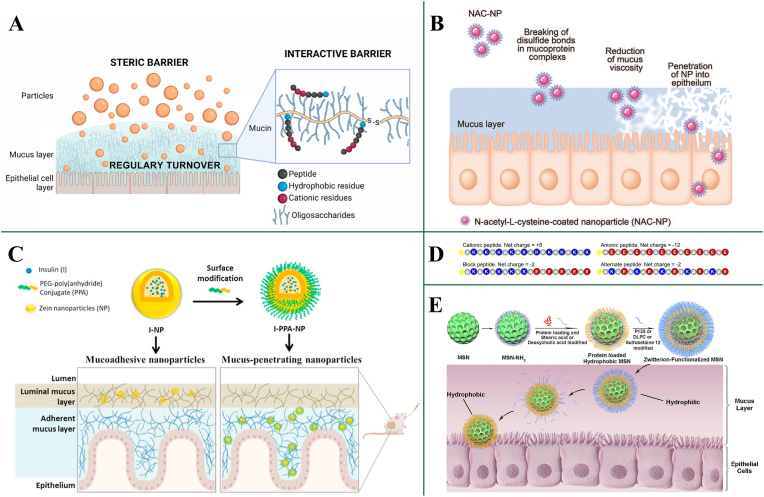
(A) Schematic diagram of the mucus barrier and its role as a steric and interactive barrier to oral protein and peptide delivery [43]. Copyright 2023 Elsevier. (B) N-acetyl-L-cysteine-coated nanoparticles can penetrate mucus effectively by breaking disulfide bonds in mucoprotein complexes and reducing the mucus viscosity [66]. Copyright 2022 Sage. (C) PEGylated surface modification promotes the penetration of nanoparticles through the mucus layer [67]. Copyright 2021 Elsevier. (D) A suite of peptides of equal molecular weight but with different net charge and spatial charge distributions are used as probes to test mucin barrier permeability [68]. Copyright 2013 Cell. (E) Schematic diagram of the preparation and enhanced permeation through mucus of the zwitterion-functionalized mesoporous silica nanoparticles [69]. Copyright 2021 Elsevier. PPA: PEG-poly(anhydride) conjugate; I-NP: insulin-loaded nanoparticles; I-PPA-NP: insulin-loaded into PPA-coated nanoparticles; MSN: mesoporous silica nanoparticles; MSN-NH_2_: amino modified MSN; P123: Pluronic P123 (EO_20_PO_70_EO_20_, M_W_ ∼ 5800); DLPC: dilauroylphosphatidylcholine.

A stronger electrostatic interaction is likely between mucin and PPs or particles, partly due to the highly negative charge of mucin, which results from the glycosylation of serine and the presence of threonine and proline domains [63]. Additionally, mucins can function as a molecular sieve, restricting the movement of larger molecules like proteins, attributed to their brush-like structural matrix [64]. Furthermore, the non-covalent interaction of mucin fibers with PPs or particles via van der Waals forces, electrostatic interactions, hydrogen bonding, and hydrophobic interactions, may lead to entrapment of PPs and particles [65], consequently impeding their absorption [43], as depicted in Fig. 2A. Although mucus presents a challenging barrier for oral PPs, to date, there are two effective strategies that have been developed to overcome this barrier: (1) Mucus penetration. (2) Mucoadhesion.

#### 3.2.1 Mucus penetration

Mucus penetration involves strategies designed to navigate through or bypass the mucus barrier to ensure that drugs reach the underlying epithelial cells. The ability to penetrate mucus is crucial for enhancing PPs bioavailability, as the mucus layer can trap and slow the diffusion of PPs molecules, thereby reducing their therapeutic effectiveness. Innovations in drug delivery systems have led to several approaches that improve mucus penetration, facilitating better absorption and efficacy of oral medications. These approaches can be broadly categorized into six strategies: (1) addition of mucolytic agents; (2) hydrophilic/slippery modification; (3) surface charge adjustment; (4) size optimization; (5) shape optimization; (6) rigidity adjustment; (7) Micro-/Nanomotors.

##### 3.2.1.1 Addition of mucolytic agents

Mucolytic agents realize the efficient penetration of mucus of oral PPs by direct disruption of the mucus layer itself. N-acetylcysteine (NAC), a member of the sulfhydryl compound class [66], possesses a free sulfhydryl group, facilitating the formation of disulfide bonds with cysteine residues within mucin subunits. This interaction leads to the cleavage of intermolecular disulfide bridges within the mucin network, thereby diminishing the extent of crosslinking in mucus gels. The resultant reduction in mucus gel density allows for improved penetration of PPs or delivery system through the mucus layer (Fig. 2B). Similar small molecule compounds like thioglycolic acid-octylamine, thiobutylamidine-dodecylamine, dithiothreitol, nacystelyn and methyl 6-thio-6-deoxy-a-D-galactopyranoside have also been explored and found similar mucolytic capability as N-acetylcysteine [70]. Except for the mucolytic compounds, mucolytic enzymes have also been immobilized on the surfaces of nanoparticles to facilitate mucus penetration. Papain-, bromelain- and trypsin-conjugated polymeric nanoparticles have been shown to increase mucosal permeation by approximately 2–3-fold in *ex vivo* mucus models, mainly through localized cleavage of the mucin network [71]. Furthermore, bromelain-decorated nanoscale liposomes significantly enhanced intestinal mucus permeation and oral absorption in preclinical models, confirming the potential of proteolytic enzymes to facilitate nanoparticle transport across the mucus barrier [72]. However, the widespread application of mucolytic agents is limited. Since the disruption of the mucus layer, while enhancing the diffusion of drugs or drug-loaded nanoparticles towards the epithelial surface, concurrently elevating the risk of pathogen dissemination and subsequent infection of epithelial cells.

##### 3.2.1.2 Hydrophilic/slippery modification

The gel-forming properties of mucus come from its important component mucins. There are a large number of hydrophobic fragments in mucin fibers, so that the hydrophobic interaction with mucins will trap PPs or delivery systems within the mucus layer [73]. The hydrophobic interaction necessitates the consideration of hydrophilic/slippery modification of nanoparticles for effective mucus penetration. Enhanced hydrophilicity is advantageous for mucus permeability, as it reduces the likelihood of interactions between PPs/nanoparticles and mucins within the mucus during local delivery. A common approach to increase hydrophilicity involves engineering a surface coating on nanoparticles through conjugation or adsorption with hydrophilic substances.

As a hydrophilic and neutrally charged polymer, PEG forms a steric hydrophilic corona that shields the hydrophobic core and minimizes adhesive interactions with mucins. Martı′nez-Lo′pez et al. [67] developed zein nanoparticles coated with PEG-poly (anhydride) conjugates, which exhibited a size of 260 nm, a negative surface charge and an insulin payload of 77 mg/mg (Fig. 2C). PEGylation significantly increased particle transport rates in the *ex vivo* porcine intestinal mucus to 20-fold higher than bare nanoparticles. *In vivo* biodistribution study conducted on rat models, also demonstrated that the bare nanoparticles were predominantly trapped within the mucus layer. In contrast, PEGylated nanoparticles (NPs) were able to penetrate through the entire mucus and reach the epithelial surface.

Notably, both the molecular weight and surface grafting density of PEG are critical determinants of the diffusion behavior of PEGylated nanoparticles across the mucus barrier [70]. Studies have demonstrated that densely grafted PEG chains with intermediate molecular weights (approximately 2–5 kDa) confer a muco-inert surface, whereas longer chains (e.g., ≥10 kDa) or insufficient grafting densities can lead to entanglement within the mucus mesh or exposure of the hydrophobic core, respectively. These effects convert PEGylated nanoparticles from mucus-penetrating to mucus-adhesive and ultimately reduce their transport efficiency through the mucus layer.

In addition, hydrophilic polymers like sodium alginate [74], poly(N-(2-hydroxypropyl) methacrylamide) [75], poly(2-hydroxyethyl acrylate), poly(2-ethyl-2-oxazoline), or poly(N-vinyl pyrrolidone) [76], poly(acrylic acid)-cysteine-6-mercaptonicotinic acid [77], polysarcosine, and zwitterionic polymers like polydopamine [78], poly-(carboxybetaine) [79], amino acid-derived polymers, polybetaines and zwitterions like betaine and phosphorylcholine [80] have also been used to modify nanoparticles or PPs to promote their penetration through the mucus layer.

##### 3.2.1.3 Surface charge adjustment

The primary functional entities within the mucus matrix are mucin glycoproteins, which are present at a concentration ranging from 1 % to 5 %. These mucins are characterized by their oligosaccharide chains, which are extensively modified with terminal sialic acid residues and sulfate groups, thereby imparting a significant negative charge to the mucin structure [21]. The preponderance of negatively charged domains within mucin fibrils results in a propensity for electrostatic interactions with positively charged nanomaterials, leading to their immobilization within the mucus. Conversely, an excessive negative charge can impede diffusion through the mucus layer due to repulsive electrostatic forces [81]. Consequently, maintaining a moderate charge is crucial for facilitating rapid diffusion of nanoparticles through the mucus barrier.

Drawing inspiration from the mechanisms employed by mucus-penetrating viruses, researchers have developed nanomaterials with balanced positive and negative charges, achieving virus-like surface properties. Surface-engineered ovalbumin protein nanoparticles (PNPs) with different surface charges were used to investigate their transport in mucus [81]. Negatively charged bare PNPs (zeta potential ≈ −23 mV) showed limited movement and remained largely confined near the entrance of native porcine nasal mucus. After PEGylation, the surface charge was shifted toward a near-neutral, mildly negative value (around −9 to −10 mV), which markedly reduced mucin binding and resulted in the fastest diffusion and deepest penetration through mucus channels. In contrast, strongly cationic PNPs (zeta potential around +20 mV) were rapidly immobilized within the mucus network due to electrostatic attraction to the negatively charged mucin fibers. These findings indicate that nanoparticles with near-neutral, weakly negative surface charge exhibit superior mucus penetration compared with strongly charged counterparts. Combination of cations and anions, like cationic octa-arginine (R8) and anionic phosphoserine (Pho), cationic cell-penetrating peptide KLPVM and anionic glutaric anhydride [82], cationic peptide (trimethyl chitosan (TMC) CSKSSDYQC) and anionic substance (γPGA-FOY), positively charged chitosan copolymer and negatively charged hyaluronic acid [83], is going to render the nanoparticles with a net neutral charge.

Except for the charge amount, the spatial arrangement of charges also influences nanomaterials’ transit within mucus. Ribbeck and colleagues explored the behavior of two peptides with identical net charges but differing charge distributions [68]. The study involved block peptides with sequences of 5 positively charged amino acids followed by 5 negatively charged ones (Fig. 2D), interspersed with alanine spacers. Peptides with a sequence of alternating positive and negative charges were synthesized. The transport behavior of these peptides within mucus was investigated using a microfluidic device. At intermediate ionic strengths (20 mM NaCl), block peptides exhibited superior transport compared to alternate, anionic, and cationic peptides. The transport of cationic peptides was significantly reduced due to electrostatic interactions with mucins, whereas the transport of anionic peptides was unaffected. Conversely, the transport of alternate peptides remained consistent irrespective of mucin presence, exhibiting a monotonically descending transport profile through the mucin barrier. In contrast, a distinct peak in the transport profile was observed for block peptides, indicating enhanced transport rates within mucins compared to water. These findings highlight that nanoscale variations in spatial charge configuration on peptide surfaces markedly impact the rate and profile of mucus diffusion. Furthermore, the ionic strength within the mucin network notably affects transport specificity, which is dependent on the detailed spatial charge arrangement. The transport of alternate peptides remained unaffected by ionic strength variations, while block peptides showed optimal transport at an intermediate ionic strength.

Uniform modification of the surface with equal positive and negative charges is crucial for the passage of nanoparticles through mucus. Therefore, electrically neutral zwitterions have become preferred modification for nano-delivery system, because they share multiple features in common with viruses, mainly are the equal and uniformly distributed positive and negative charges. Han et al. [80] used zwitterionic betaine polymer to modify micelles to achieve a virus-mimetic surface, which accomplish much faster diffusion rates in mucus compared with the cationic poly((2-(methacryloyloxy) ethyl) trimethylammonium chloride), anionic poly(3-sulforpropyl methacrylate) potassium salt and neutral nonionic PEG modified nanoparticles. Gao et al. [69] devised a zwitterion-functionalized mesoporous silica nanoparticles (MSNs) for enhancing oral administration of insulin (Fig. 2E). Surface modified zwitterion-sulobetaine 12 significantly promoted the penetration of nanoparticles through the mucus layer, compared to unmodified aminated MSNs. In addition, dilauroylphosphatidylcholine, phosphorylcholine, poly(sulfobetaine), poly(carboxybetaine), poly(phosphobetaine), and polydopamine [78] can also achieve virus-like zwitterionic modifications on the surface of nanoparticles for improved penetration through mucus.

In addition to using an equal amount of positive and negative charge modification strategy similar to virus surfaces, non-charged polymer modification is also an effective method to prevent nanoparticles from being captured or repelled by negatively charged mucins. Non-charged polymers like low molecular weight PEG [76], poly(2-alkyl-2-oxazolines), polysarcosine, poly(vinyl alcohol), polyglycidols, poly(2-hydroxyethylmethacrylate), poly(2-hydroxyethylacrylate), polyglycidols, poly-(N-(2-hydroxypropyl)methacrylamide) and low molecular weight dextran have also been being used to overcome the electric adsorption or repulsion of nanoparticles by mucins, facilitating the transit of nanoparticles through the mucus.

##### 3.2.1.4 Size optimization

Mucin, the primary structural component of mucus, assembles into a dense fibrous mesh that acts as a size-selective barrier to foreign particles. The mesh spacing varies with anatomical site, with pore sizes typically ranging from ∼40 nm to several micrometers [84]. To minimize steric obstruction by this network, nanocarriers must be dimensionally optimized relative to the mucus mesh. Bandi et al. [85] prepared fluorescent polystyrene nanoparticles with diameters of 50, 100, 200, 500, 750 and 1000 nm to investigate the impact of particle size on mucus penetration. Smaller nanoparticles (50 and 200 nm) permeated across mucus to a significantly greater extent (p < 0.05) than larger counterparts (500 and 750 nm). Similarly, He et al. [86] compared the diffusivity of nanocrystals of different sizes in simulated mucus and found that particles with a smaller diameter (∼250 nm) exhibited higher mean squared displacements than larger ones (535 and 1100 nm). Above all, the diffusion rate of NPs is greatly influenced by the mesh pore structure of the mucus, which highlights the crucial impact of particle size on penetration efficiency through the mucus layer.

##### 3.2.1.5 Shape optimization

In addition to particle size, the shape of nanoparticles also plays a significant role in their ability to penetrate the mucus layer. Research has elucidated the correlation between microbial morphology and motility within mucus. The helical shape of *Helicobacter pylori* has been identified as a key factor in facilitating its traversal through the gastric mucus layer, with its shape providing enhanced movement efficiency compared to other forms [87]. This realization of the impact of shape on bacterial locomotion has catalyzed the advent of shape modulation as an innovative strategy to surmount the mucus barrier. Iriarte-Mesa et al. [88] designed bioinspired MSNs with distinct morphologies including spherical dendritic mesoporous silica nanoparticles (D90, D130), virus-like particles (VlNPs) and rod-shaped nanorods (NrNPs, ∼160 × 35 nm), to investigate their behavior at an intestinal mucus barrier. In a Caco-2/HT29-MTX-E12 co-culture covered by native mucus, rod-shaped NrNPs exhibited the greatest ability to traverse the mucus layer, showing the largest focal plane shift toward the cell monolayer (≈45 μm) compared with D90 spheres (36 μm) and VlNPs (32 μm). Similar penetration was only observed for much smaller 35 nm spheres. The superior mucus transport of NrNPs is attributed to their small cross-sectional diameter and rod-specific rotational dynamics within the mucus mesh, which together facilitate deeper penetration through the mucus barrier. Meanwhile, Bao et al. [89] synthesized a series of peptosomes of varied dimensions, shapes, and structural rigidities through the self-assembly of amphiphilic α-lactalbumin peptides to study the mucus penetration. Short nanotubes demonstrated superior mucus permeation capabilities, compared with the long nanotubes, big nanospheres, small nanospheres, and crosslinked short nanotubes. The underlying mechanism for the enhanced mucus permeability of short nanotubes was also elucidated using coarse-grained molecular dynamics simulations, which revealed that brief jiggling movements and the capacity to traverse mucus network pores were instrumental. Therefore, the shape of nanorods is more suitable for penetrating the mucus layer.

##### 3.2.1.6 Rigidity adjustment

Rigidity is another critical factor that can be adjusted to influence the penetration of particles within mucus [90]. Drawing inspiration from the crucial role of elasticity in the translocation of cancer cells and red blood cells through constricted spaces *in vivo*, researchers hypothesized that the strategic modulation of nanoparticle rigidity could enhance their ability to penetrate mucus. Yu et al. [91] synthesized poly(lactic-co-glycolic acid) (PLGA)-lipid NPs of varied rigidities, including soft, semi-elastic, and hard variants, and assessed their mucus penetration capabilities. It was observed that NPs possessing a moderate rigidity (∼50 MPa) demonstrated superior diffusion properties in intestinal mucus. Specifically, orally administered semi-elastic NPs exhibited a notable increase in the bioavailability of the loaded drug, surpassing that of both soft and hard nanoparticle formulations. Molecular dynamics simulations alongside Single Molecule Real-time imaging were employed to elucidate the mechanisms, revealing that semi-elastic NPs underwent deformation into ellipsoidal shapes, which facilitated rapid penetration through rotation.

**Fig. 3:**
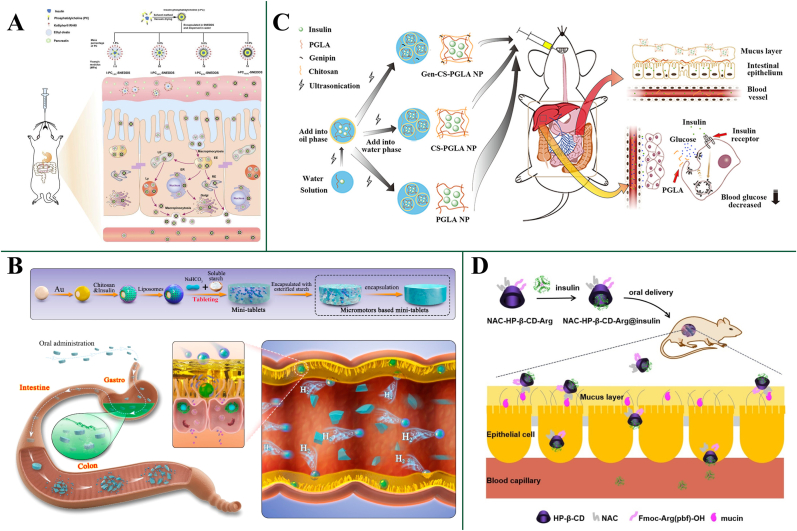
(A) Schematic diagram of the SNEDDS of varied rigidities and their different mucus penetration and cellular uptake [91]. Copyright 2023 Elsevier. (B) Illustration of micromotor based mini-tablet system and the motion of the released micromotors promoted the enhanced mucosa penetration and insulin delivery in the colon [95]. Copyright 2023 American Chemical Society. (C) Mucoadhesive chitosan coating, enhanced by genipin cross-linking, allowing nanoparticles for prolonged intestinal residence time and improved oral absorption of insulin [96]. Copyright 2017 American Chemical Society. (D) Thiolated N-acetyl-L-cysteine modification makes the complex strongly bound with mucin [97]. Copyright 2021 Elsevier.

Conversely, rigid NPs lacked the ability to deform, and soft NPs encountered impediments due to interactions with mucin fibers. Li and co-workers presented self-nanoemulsifying drug delivery systems (SNEDDS) with varying mechanical rigidity, achieved by adjusting the phosphatidylcholine content (Fig. 3A) [92]. Their study also confirmed that the rigidity of SNEDDS significantly influences their ability to penetrate the mucus layer. Harder SNEDDS exhibited superior mucus penetration capabilities, with up to twice the fluorescence intensity in mucus layers compared to the softer counterparts. Additionally, Yu et al. [93] fabricated an array of liposomes with varying phase transition temperatures and evaluated their mucus diffusivity at different ambient temperatures. Findings indicated that liposomes achieved optimal rigidity and permeability when the ambient temperature approximated their phase transition temperatures, attributed to their ability to deform into ellipsoids more efficiently at temperatures close to the Tm, such as body temperature, thereby enhancing their mobility and facilitating rapid diffusion. Complementarily, Zheng et al. [94] reported a decrease in the mean squared displacements of NPs in mucus with rising elasticity, further highlighting the intricate relationship between NPs rigidity and mucus permeability.

In summary, rigidity plays a crucial role in determining the efficiency of NPs penetration through mucus layers. Research has demonstrated that while increased rigidity can enhance mucus penetration, overly rigid NPs may lack the necessary deformability to navigate effectively through the mucus network. Conversely, NPs that are too soft may encounter difficulties due to interactions with mucin fibers, which impede their mobility. Optimal mucus penetration is typically achieved with NPs that possess moderate rigidity, allowing them to deform into appropriate shapes under physiological conditions, thereby facilitating more efficient diffusion through the mucus. Therefore, a careful balance between rigidity and deformability is essential for designing NPs that can successfully realize mucus penetration and improve oral PPs delivery efficacy.

##### 3.2.1.7 Micro-/nanomotors

Micro-/nanomotors represent an innovative strategy for actively penetrating the mucus layer, contrasting with the passive diffusion methods discussed earlier [98]. These tiny machines can self-propel through biological fluids, providing a means to overcome the physical barrier of mucus more effectively. The active propulsion allows for timely and targeted penetration, avoiding the periodic clearance mechanisms that often remove foreign substances from the mucus layer. Micro-/nanomotors achieve propulsion through various mechanisms, including catalytic reactions, external fields (magnetic, electric, or ultrasound), or light-driven processes [98]. This active movement enables nanoparticles to navigate the viscoelastic environment of mucus, facilitating deeper penetration and enhanced delivery of PPs to the underlying epithelial cells. Liu and co-workers developed a micromotor-based mini-tablet system for the oral delivery of insulin, demonstrating significant advancements in overcoming the GI barriers, particularly the mucus layer (Fig. 3B) [95]. The micromotors, composed of magnesium microparticles with a nanogold layer and coated with chitosan and liposomes, were capable of self-propulsion through the production of hydrogen bubbles upon reaction with water, achieving speeds up to 70.9 μm s^−1^ in simulated colon fluid containing 8 mg mL^−1^ mucin. This autonomous movement allowed the micromotors to effectively penetrate the mucus layer, reducing the resistance encountered in the viscous environment and promoting deeper mucosal penetration. Micromotors' motion enabled enhanced mucus penetration and facilitated the cellular uptake of insulin, as evidenced by the stronger fluorescence observed in Caco-2 cells incubated with insulin-loaded micromotors compared to passive insulin-loaded SiO_2_ microparticles. This active propulsion mechanism significantly improved the bioavailability and therapeutic efficacy of orally administered insulin, maintaining stable blood glucose levels in diabetic rats for over 5 h. In summary, micro-/nanomotors offer a promising and innovative approach to overcoming the mucus barrier for oral delivery of PPs, providing an active delivery mechanism that enhances therapeutic efficacy and bioavailability.

#### 3.2.2 Mucoadhesion

Except for mucus penetration, mucoadhesion is also a common strategy to overcome the mucus barrier [99]. While mucus penetration focuses on passing through the mucus layer, mucoadhesion leverages the mucus as a beneficial barrier rather than an obstacle. Mucoadhesion refers to the strategy where drug delivery systems are designed to adhere to the mucus layer, thereby prolonging the residence time of the therapeutic agents in the GI tract [100]. This extended residence time can enhance drug absorption by maintaining a higher local concentration of the therapeutic agent at the absorption site and protecting it from enzymatic degradation. Thus, although it may seem contradictory, both mucus penetration and mucoadhesion can serve as effective strategies for improving the oral bioavailability of PPs, each addressing different aspects of the mucus challenge. Mucoadhesion was first observed by Florey in 1962 with India ink particles adhering to intestinal mucus [101]. Most microparticles or nanoparticles exhibit non-specific mucoadhesion with intestinal mucus. The mucoadhesive properties of particles are significantly influenced by their hydrophobicity, surface charge, and chemical structure [100]. This phenomenon occurs because the particles primarily interact with the mucins in the mucus layer, which are hydrophobic, negatively charged, and contain sulfhydrylated glycoproteins. Considering the structural characteristics of mucins, particles can be specifically modified to enhance mucoadhesion. Strategies for such modifications include hydrophobic modification, positively charged modification, and thiolation. Hydrophobic modification enhances interactions with the hydrophobic regions of mucins, positively charged modification utilizes electrostatic attraction to the negatively charged mucins, and thiolation involves the formation of disulfide bonds with the sulfhydrated components of mucins.

Last but not least, it is important to note that particle size also significantly affects mucoadhesion [100]. To achieve effective mucoadhesion, particles have to penetrate the frequent turnover of the loosely adherent mucus layer and reach the firmly adherent mucus at the bottom [102]. Otherwise, the frequent turnover of the loosely adherent mucus layer will result in direct clearance of the particles adhered to it. The mucin network serves as a steric barrier, forming a size-exclusion filter. Electron microscopy measurements have shown that the average mesh-spacing of intestinal mucus is ∼200 nm. If nanoparticles are too large, they cannot penetrate the loosely adherent mucus layer and reach the firmly adherent mucus. Studies have shown that smaller particles tend to adhere more effectively to mucus than larger particles [100].

##### 3.2.2.1 Hydrophobic modification

Mucins possess hydrophobic domains, including the exposed segments of their protein backbone chains, demonstrating a strong affinity for hydrophobic materials. Sonia and colleagues developed hydrophobic chitosan microparticles for oral insulin delivery by N-acylation with octanoyl and oleoyl chloride. The modification enhanced mucoadhesion through hydrophobic interactions between the hydrophobic moieties of chitosan and the hydrophobic components of mucin glycoproteins. Contact angle measurements showed that oleoyl chitosan had a higher contact angle (112°) compared to octanoyl chitosan (90°), indicating greater hydrophobicity. Mucoadhesion studies revealed that oleoyl chitosan was more mucoadhesive, adhering better to intestinal mucus due to these interactions. *In vivo* uptake studies demonstrated that oleoyl chitosan microparticles exhibited significantly higher uptake in rat intestinal Sections compared to octanoyl chitosan. This increased mucoadhesion results in prolonged residence time of microparticles in the GI tract, protecting insulin from enzymatic degradation and improving its absorption, thus enhancing the bioavailability of orally delivered insulin. Reboredo et al. [103] prepared bare zein nanoparticles as oral insulin carriers to enhance insulin oral bioavailability by mucoadhesion. Bare zein nanoparticles significantly enhanced the retention of nanoparticles within the mucus layer through hydrophobic interactions and hydrogen bonding with the mucin glycoproteins. Overall, these studies underscore the potential of hydrophobic modification to improve the mucoadhesion and pharmacological effectiveness of oral PPs formulations.

##### 3.2.2.2 Positively charged modification

Due to the negative charge of the mucin in the mucus layer, positively charged particles exhibit strong mucoadhesive properties. Cationic chitosan, particularly N-trimethyl chitosan, is commonly used to engineer or coat nanoparticles to enhance mucoadhesion through electrostatic interactions with mucins. Cheng et al. [99] coated insulin-loaded poly (n-butylcyanoacrylate) nanoparticles with cationic chitosan, significantly extending the retention time of the nanoparticles in the mucus layer, since the electrostatic interaction between the positively charged chitosan and the negatively charged mucin. *In vivo* studies revealed that the mucoadhesive cationic nanoparticles exhibited a sustained hypoglycemic effect in diabetic rats, with a pharmacological availability of 8.44 %. Zhang and co-workers presented genipin (Gen)-CS-PGLA nanoparticles, which exhibited enhanced mucoadhesion and improved oral insulin bioavailability (Fig. 3C) [96]. These nanoparticles were designed to leverage the mucoadhesive properties of CS, enhanced by genipin (Gen) cross-linking, allowing for prolonged intestinal residence time. *In vivo* experiments with type 1 diabetic rats showed that Gen-CS-PGLA nanoparticles resulted in a maximal blood glucose depression of 30 % at 6 h after administration, compared to 32 % at 4 h for CS-PGLA nanoparticles and 32 % at 2 h for PGLA nanoparticles. The delayed but sustained hypoglycemic effect observed with Gen-CS-PGLA nanoparticles can be attributed to the genipin-induced cross-linking, which enhances the structural stability and mucoadhesive properties of the nanoparticles. This stability ensures a more controlled and gradual release of insulin, leading to prolonged intestinal absorption and a sustained hypoglycemic effect. The improved mucoadhesion facilitates a longer residence time in the intestinal tract, allowing for more efficient insulin absorption over time.

In addition, Sheng et al. [104] formulated insulin-loaded N-trimethyl chitosan chloride-coated polylactide-co-glycolide (PLGA) nanoparticles (Ins TMC-PLGA NPs). These TMC-coated nanoparticles exhibited a strong positive zeta potential (45.2 ± 4.6 mV) and had a particle size of 247.6 ± 7.2 nm. Compared with unmodified PLGA NPs, the positively charged TMC-PLGA NPs demonstrated improved mucoadhesion, resulting in enhanced mucus adhesion in mucus-secreting HT29-MTX cells. At the same time, TMC-PLGA NPs also increased cellular uptake of insulin via clathrin- or adsorption-mediated endocytosis in Caco-2 cells, and improved insulin permeation across a Caco-2 cell monolayer by opening tight junctions. Following oral administration in mice, TMC-PLGA NPs demonstrated slower GI transit compared to unmodified PLGA NPs, indicating enhanced mucoadhesive properties due to TMC coating. In pharmacological studies with diabetic rats, orally administered Ins TMC-PLGA NPs produced a stronger hypoglycemic effect, exhibiting a 2-fold increase in relative pharmacological bioavailability compared to unmodified NPs. These studies highlight the ability of positively charged nanoparticles to significantly increase the residence time of nanoparticles in the mucus layer, thereby enhancing insulin absorption and providing sustained therapeutic effects. However, the variation in the observed bioavailability across different formulations suggests that factors such as particle size, zeta potential, and the extent of cross-linking play crucial roles in determining the overall efficacy of these systems.

##### 3.2.2.3 Thiolation

Thiolation, or the addition of thiol groups to the surface of oral PPs delivery system, is another common strategy to enhance mucoadhesive ability. This method promotes the formation of disulfide bonds between the thiol groups of the delivery system and the cysteine-rich subdomains of mucus glycoproteins. Li et al. [97] developed a novel N-acetyl-L-cysteine and arginine modified hydroxypropyl-β-cyclodextrin (NAC-HP-β-CD-Arg) for oral insulin delivery (Fig. 3D). The thiol groups in NAC-HP-β-CD-Arg form disulfide bonds with cysteine-rich subdomains of mucus glycoproteins, enhancing mucoadhesion and prolonging the residence time of the prepared inclusion complex on the intestinal mucosa. This complex showed an 8-fold increase in insulin permeability across Caco-2 cell monolayers compared to free insulin. In diabetic rats, the orally administered NAC-HP-β-CD-Arg@insulin complex achieved a bioavailability of 9.06 %, compared to 0.3 % for free insulin, and provided a sustained hypoglycemic effect, maintaining reduced blood glucose levels for 24 h. Thiolation significantly enhances the mucoadhesion of oral PPs delivery systems or complex by forming disulfide bonds with mucus glycoproteins, greatly improving the bioavailability and effectiveness of oral PPs.

#### 3.2.3 Comparison of mucus penetration and mucoadhesion

Mucus penetration and mucoadhesion are two critical strategies for overcoming the GI mucus barrier, each with distinct mechanisms and implications for oral PPs absorption. Mucus penetration focuses on the traversal of drug delivery systems through the mucus layer to directly interact with the underlying epithelial cells. This strategy improves the efficiency of drug delivery by bypassing the mucus barrier, enabling nanoparticles to move freely and reach the target cells more effectively. Surface modifications with hydrophilic polymers, such as PEGylation, are commonly employed to achieve mucus penetration. Mucus-penetrating nanoparticles reduce impact of mucus turnover, providing a more consistent absorption profile. However, if there is no adhesion between mucus and nanoparticles, they might be cleared away by mucus turnover due to their back-diffusion, leading to rapid clearance from the GI tract before the drug can be absorbed. Therefore, careful optimization of surface properties is required to balance penetration efficiency and mucoadhesion, ensuring effective drug delivery.

In contrast, mucoadhesion involves the adherence of drug delivery systems to the mucus layer covering the epithelial cells of the GI tract. This strategy prolongs the residence time of the drug at the absorption site, potentially increasing the local concentration and facilitating greater drug uptake. The use of mucoadhesive materials, such as chitosan, allows for the formation of electrostatic interactions, hydrogen bonds, and van der Waals forces with the mucus components. This approach is particularly advantageous for sustained-release formulations, as it provides a controlled and extended drug release profile, and enhances drug stability by protecting it from enzymatic degradation in the GI tract. However, mucoadhesive systems may face challenges such as entrapment in the loosely adherent mucus layer, leading to clearance from the GI tract before the PPs can be absorbed. Additionally, variability in mucus turnover rates can affect the consistency of drug delivery and absorption, posing a limitation to the overall efficacy of mucoadhesive formulations.

### 3.3 Strategies for overcoming intestinal epithelial barrier

The intestinal epithelium located beneath the mucus is another significant barrier to the delivery of oral PPs. The epithelial cells are a single layer of cells lining the gut lumen, which serve as both a barrier for foreign substances and a facilitator of nutrient absorption. The epithelium is primarily made up of several cell types, including enterocytes, goblet cells, Paneth cells, enteroendocrine cells, dendritic cells and M cells, each contributing uniquely to the intestine's functions. Enterocytes are the most abundant cell type in the intestinal epithelium, which are specialized for nutrient absorption, featuring microvilli on their apical surface that form the brush border, significantly increasing the surface area for absorption [105]. They actively and passively transport nutrients, electrolytes, and water from the gut lumen into the bloodstream. Goblet cells secrete mucus, a viscous fluid composed primarily of mucins, that lubricates the intestinal contents and forms a protective layer over the epithelium, defending against mechanical damage, pathogens, and digestive enzymes. Paneth cells locate at the base of the intestinal crypts, which secrete antimicrobial peptides and proteins, such as lysozyme and defensins, contributing to the maintenance of the intestinal microbiome and protection against microbial invasion [106]. Enteroendocrine cells produce various hormones in response to stimuli from the gut lumen, such as nutrients or bacterial products. These hormones regulate digestive processes, including enzyme secretion, gut motility, and appetite [107]. Dendritic cells play a crucial role in maintaining immune homeostasis in the intestine by integrating humoral and cellular immune responses. M cells are specialized for the transcytosis of antigens and pathogens from the gut lumen to immune cells in the underlying lymphoid tissue, playing a critical role in initiating immune responses and maintaining gut homeostasis.

Meanwhile, the epithelial cells are closely joined by tight junctions, which control the paracellular pathway between cells. These tight junctions are effective at preventing the passage of large molecules, including proteins, from the apical gut lumen into the basal intestinal space through intercellular space [108]. These different cells and tight junction proteins between cells form the intestinal epithelium, effectively blocking the entry of foreign substances. Although the physiological architecture of IECs renders them a formidable barrier rejecting the entry of any oral PPs, to date, there are several effective strategies that have been developed to overcome this barrier: (1) Permeation enhancers, (2) Chemical modification, (3) Nanoparticle-based delivery systems.

#### 3.3.1 Permeation enhancers

Permeation enhancers represent a class of excipients that can transiently modify the epithelial architecture, thus significantly boosting intestinal permeability of co-administered payloads like PPs through both transcellular and paracellular pathways. The interest in permeation enhancers surged following a 1961 study demonstrating that EDTA could significantly increase the oral uptake of heparin in dogs [109]. Advances have led to the development of various natural, semi-synthetic and synthetic permeation enhancers, including solvents (e.g. ethanol), chelating agents, surfactants, ionic liquids (choline and organic acids [110]) endogenous secretions like bile salts, as well as high molecular weight polymers (e.g. chitosan or alginate [99]), bacterial toxins like zona occludens toxin [111] and cell-penetrating peptides (CPPs). These enhancers improve the oral bioavailability of PPs by facilitating transport across epithelial barriers either via paracellular routes, by opening tight junctions, or through transcellular pathways, by enhancing membrane permeability, or through a synergistic combination of both these mechanisms. However, the paracellular route is not a viable pathway for PPs with a larger size, as the intercellular space ranges between 10 and 30–50 Å, which is insufficient to accommodate their size [112].

Chelating agents such as EDTA, Ethylene glycol-bis(β-aminoethyl ether)-N,N,N′,N′-tetraacetic acid ‌ (EGTA), diethylene triamine pentaacetic acid (DTPA) and citric acid typically facilitate paracellular absorption by opening tight junctions. This modulation is attributed to their ability to chelate extracellular metal ions, thereby reducing intracellular metal ions levels and transiently loosening tight junctions [113]. EDTA is proposed to enhance paracellular transport by chelating extracellular Ca^2+^, thereby disrupting epithelial tight junction integrity and increasing epithelial permeability [51]. Similarly, DTPA exerts dual functions by nonspecifically chelating divalent metal ions (Ca^2+^, Mg^2+^, Zn^2+^), leading to both transient tight junction opening and inhibition of intestinal protease activity. EGTA, while structurally related to EDTA, demonstrates even higher selectivity and binding affinity for Ca^2+^, thereby inducing a more pronounced modulation of tight junction permeability.

**Fig. 4:**
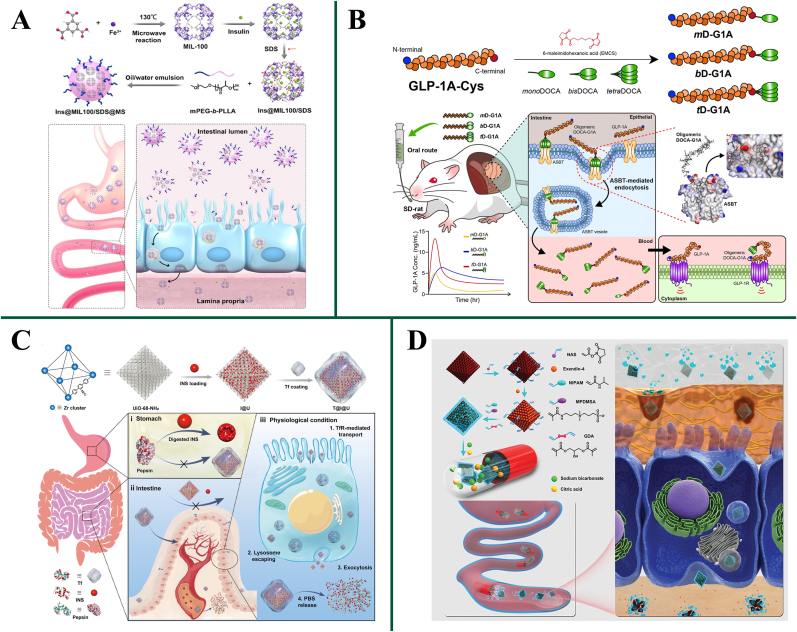
(A) Dissolution of the Ins@MIL100/SDS@MS microspheres in the intestine renders exposure and penetration through the intestinal epithelium of Ins@MIL100/SDS by SDS modification [115]. Copyright 2020 American Chemical Society. (B) GLP-1A was conjugated with oligomeric DOCAs to improve its oral bioavailability by utilizing transporter-mediated endocytosis [116]. Copyright 2023 Springer Nature. (C) The transferrin-coated UiO-68-NH_2_ nanosystem contributed to overall intensive intestinal cell absorption [117]. Copyright 2022 Science. (D) The zwitterionic surface modification of the Ex@MIL101@Gel ± nanoparticles helps the transportation across the epithelial layer of the intestine [118]. Copyright 2021 Wiley.

Surfactants, particularly sodium caprylate/caprate and endogenous bile salts, are pivotal permeation enhancers in clinical settings [114]. SNAC, a sodium caprylate derivative, can not only protect semaglutide from degradation by gastric protease, but also significantly improves the GI transport of simeglutide [10]. The majority of research suggest that the hydrophobic SNAC, when non-covalently bound to peptides, facilitates their absorption through the intestinal epithelium via transcellular pathways [111]. Sodium dodecyl sulfate (SDS) also serves as a crucial permeation enhancer. Zhou and co-workers developed a novel nanocomposite vehicle incorporating SDS-modified MOF nanoparticles (MIL100/SDS), which significantly enhanced the oral absorption of insulin by promoting its permeation across IECs (Fig. 4A) [115]. The SDS modification of the MOF nanoparticles significantly boosted the cellular uptake of insulin, since SDS facilitates the interaction of nanoparticles with IECs. The SDS-coated Ins@MIL100/SDS nanoparticles exhibited a higher internalization rate in Caco-2 cells compared to unmodified nanoparticles, leading to a more efficient transcellular transport. Furthermore, *in vivo* pharmacokinetic analyses revealed that the oral administration of Ins@MIL100/SDS@MS (microspheres containing SDS-modified nanoparticles) in type 1 diabetic rat models resulted in a significant reduction in blood glucose levels, with a relative pharmacological availability of 7.8 %, indicating a marked improvement in oral insulin bioavailability.

CPPs, which originate from the transactivator of transcription protein of the human immunodeficiency virus, have been shown to elevate the membrane permeability of PPs [119]. CPPs typically harbor high levels of positively charged amino acids (arginine and lysine), which enable strong electrostatic interactions with negatively charged cell surfaces. In addition, CPPs contain hydrophobic domains (e.g., tryptophan residues) that facilitate their translocation across the lipid bilayer, further enhancing intracellular delivery. Penetratin®, the first CPP, comprising of 16 amino acids (RQIKIWFQNRRMKWKK), was identified in 1994 [120]. Therapeutic PPs are either chemically conjugated to CPPs or non-covalently associated to form complexes. The proposed action mechanism of CPPs involves facilitating cellular uptake of PPs by augmenting both paracellular and transcellular pathways via the endocytic route [121]. Uhl et al. [122] developed cell-penetrating peptide (CPP)-modified liposomal nanocarriers for the oral delivery of PPs, using vancomycin and exenatide as model drugs. *In vivo* studies in Beagle dogs demonstrated that CPP–liposomes achieved a markedly improved oral bioavailability, 3.9 % for vancomycin and 0.3 % for exenatide, representing a 20-fold increase over free drug. The enhanced absorption was attributed to the protective effect of liposomal encapsulation against enzymatic degradation and the CPP-mediated permeation of intestinal epithelial monolayers. These findings indicate that CPPs can effectively enhance the oral absorption of PPs; however, nanoparticle encapsulation is essential to protect it from enzymatic degradation in the GI tract.

Although permeation enhancers have proven effective in augmenting the oral bioavailability of PPs, the toxicity caused by the entry of foreign substances into the body due to the opening of TJs is also a safety issue that we cannot ignore [111]. Safety and regulatory compliance present significant challenges in the utilization of these permeation enhancers for oral delivery of PPs. In this regard, Lamson et al. [123] developed a biocompatible plant-derived enhancer, identifying the red pigment pelargonidin from strawberries as a potent and well-tolerated intestinal permeation enhancer. In murine studies, oral co-administration of pelargonidin with insulin resulted in sustained glucose reduction for over 4 h, with bioactivity exceeding 100 % relative to subcutaneous injection. Notably, daily administration of pelargonidin for one month produced no observable adverse effects, including weight loss, tissue injury, or inflammatory responses, suggesting its potential for safe long-term use. Although, to date, no major adverse events have been consistently reported for the permeation enhancers evaluated in clinical trials, further studies and long-term follow-ups are needed to comprehensively assess their safety profiles.

#### 3.3.2 Chemical modification

The chemical modification strategy is a prevalent method for modifying the physicochemical properties of drugs through chemical derivatization, aimed at enhancing stability, solubility, or permeability. In the context of oral administration of PPs, chemical modification plays a pivotal role in overcoming the intestinal epithelial barrier. Key approaches in this strategy include lipidation, peptide cyclization and ligand modification. These strategies promote the oral absorption of PPs by improving their stability and increasing the likelihood of interactions with the intestinal epithelial cell membrane, thereby facilitating their trans-epithelial transport.

##### 3.3.2.1 Lipidation

The cell membrane is primarily composed of a phospholipid bilayer, where the hydrophobic tails of phospholipids face inward, creating a barrier that is more permeable to lipid-soluble substances. This hydrophobic nature of the membrane facilitates the passage of lipid-soluble substances, as they can easily interact with the lipid bilayer. Therefore, lipidation represents an efficient approach for chemical modification of PPs, which increases the hydrophobicity of PPs and thereby improves their permeability. By increasing the hydrophobicity of the PPs, these modifications can enhance their interaction with cell membranes, thereby improving their permeability across the intestinal epithelial barrier. Emeh et al. [124] investigated the effect of lipidation on the intestinal absorption of glucagon-like peptide-1 agonist (GLP-1A) analogues, using a series of peptides with varying degrees of lipidation: J211 (non-lipidated), J229 (mono-lipidated), MEDI7219 (bis-lipidated), and semaglutide (mono-lipidated). The degree of lipidation positively correlated with enhanced intestinal absorption. The bis-lipidated MEDI7219 showed the highest bioavailability (17.3 %), followed by J229 with a mono-lipidation (4 %), and the non-lipidated J211 exhibited the lowest bioavailability (<1 %). These findings highlight that lipidation, particularly bis-lipidation, plays a crucial role in increasing the intestinal absorption of GLP-1A analogues. The increased lipidation also contributed to improved stability against enzymatic degradation, further supporting the higher bioavailability of the lipidated peptides compared to the non-lipidated counterpart.

Moreover, other hydrophobic molecules such as cholesterol, glycosylphosphatidylinositol, phosphatidylethanolamine, prenyl groups can be conjugated to PPs to improve their oral bioavailability [38]. Despite the significant enhancement in the oral bioavailability of PPs achieved through lipidation, recent studies employing direct covalent attachment of lipophilic molecules to PPs for improving oral absorption have become increasingly scarce. This decline may be attributed to the technical complexity associated with lipidation processes or the comparatively lower efficiency of such modifications relative to alternative strategies. However, lipidation remains an important chemical modification strategy for PPs. At present, it is more commonly employed to modify parenterally administered PPs, as it not only enhances their *in vivo* stability but also prolongs their duration of action, exemplified by the FDA-approved liraglutide. Liraglutide, which is lipidized by attaching palmitic acid via a glutamic spacer, thereby exhibits a prolonged plasma half-life that increases from 2 min to 13 h [125].

##### 3.3.2.2 Peptide cyclization

Peptide cyclization is acknowledged as an effective chemical modification technique for PPs, enhancing oral peptide delivery by protecting peptides from degradation in the harsh GI environment and improving their absorption across IECs by reducing their intermolecular interactions with the aqueous solvent [126]. This strategy involves creating cyclic peptide structures that can open and close reversibly, increasing stability, reducing size, and enhancing lipophilicity, which collectively boost the peptide's bioavailability when administered orally [127]. Merz et al. [50] developed a library of small macrocyclic thrombin inhibitors by cyclizing short linear peptides via non-reducible dithioether and subsequently ring-closing metathesis-based linkers and comparing them with their linear precursors. The resulting cyclic peptides displayed several advantageous features: (1) greatly improved proteolytic stability in simulated gastric and intestinal fluids, as well as enhanced metabolic stability in rat liver microsomes; (2) optimized polarity and intramolecular hydrogen-bonding patterns that conferred high passive membrane permeability in parallel artificial membrane permeability assays, approaching that of small oral drugs; and (3) nanomolar affinity and selectivity for thrombin following structure-guided optimization of the macrocyclic scaffold. Importantly, lead macrocycles such as compound 46 achieved oral bioavailability of up to ∼18 % in rats, indicating that appropriately designed cyclic peptides can effectively traverse the intestinal epithelial barrier and reach therapeutically relevant systemic exposures.

Furthermore, MYCAPSSA™, a cyclic octreotide analog, was approved by the FDA as the first oral formulation of octreotide for the treatment of acromegaly. This approval highlights the clinical applicability of cyclic peptides in improving the bioavailability of peptide-based therapeutics. The success of MYCAPSSA™ further validates the potential of peptide cyclization in enhancing the oral delivery of biologically active peptides. Despite the numerous benefits of peptide cyclization, such as enhanced stability, increased proteolytic resistance, and improved membrane permeability, these advantages can only be realized if the peptide satisfies conditions like appropriate conformational rigidity and the ability to fold into a stable cyclic structure.

##### 3.3.2.3 Ligand modification

Intestinal epithelial cells are characterized by the high expression of various transporters and receptors, which ensure the efficient absorption of nutrients and essential substances. Leveraging this physiological feature, various nutritional components such as vitamins, saccharides, and fatty acids have been extensively studied as ligands to decorate drug molecules or carriers, facilitating receptor-mediated active transport across the epithelial barrier. This approach typically involves the conjugation of specific ligands to the PPs, enabling targeted interactions with receptors or transporters on the surface of epithelial cells. Such interactions can facilitate receptor-mediated endocytosis or transcytosis, thus promoting the transport of PPs across the intestinal epithelial barrier. Moreover, ligand modification can enhance the stability of PPs in the harsh GI environment, protect them from enzymatic degradation. Kweon and co-workers developed a chimeric GLP-1A by covalently conjugating oligomeric deoxycholic acid (DOCA) to GLP-1A using a maleimide reaction (Fig. 4B) [116]. The DOCA conjugation specifically targeted the ASBT, facilitating GLP-1A absorption across IECs by utilizing transporter-mediated endocytosis. *In vitro* studies using Caco-2 cells revealed significantly higher permeability of the DOCA-GLP-1A conjugates compared to the non-conjugated GLP-1A, indicating enhanced cellular uptake due to the DOCA modification. *In vivo* studies in rats further supported these findings, with the DOCA-GLP-1A conjugates showing a much higher oral bioavailability compared to unmodified GLP-1A. In addition, Vitamin B12 and transferrin [128] can also serve as ligands covalently grafted onto PPs, enhancing their oral absorption.

#### 3.3.3 Nanoparticle-based delivery systems

Direct chemical modification of PPs can enhance their ability to overcome intestinal epithelial barriers but often comes at the cost of reduced pharmacological efficacy due to structural changes. To address these limitations, encapsulating PPs within NPs has emerged as a preferred strategy. This approach preserves the native three-dimensional conformation and bioactivity of PPs, while surface modifications of the nanoparticles enable efficient intestinal epithelial transport. Key strategies for modifying PPs-loaded nanoparticles include hydrophobic modification, surface charge adjustment, ligand modification and zwitterionic modification.

##### 3.3.3.1 Hydrophobic surface modification of nanoparticles

As previously discussed in Section 3.3.2.1 *Lipidation*, the cellular membrane exhibits significant hydrophobicity due to its lipid bilayer structure. This characteristic can be strategically exploited by modifying nanoparticles with hydrophobic moieties, thereby increasing their affinity for the intestinal epithelial cell membrane. Enhanced interactions between hydrophobically modified nanoparticles and the cell membrane can facilitate cellular uptake through processes such as endocytosis or direct membrane fusion. Li et al. [129] devised pH-responsive copolymer nanoparticles (PLGA-*Hyd*-PEG), consisting of PEG and PLGA linked by a hydrazone bond. Upon reaching the jejunal epithelial surface (pH ∼5.5), the hydrazone bond was hydrolyzed, causing rapid cleavage of the PEG shell and converting the nanoparticle surface from hydrophilic to hydrophobic. The hydrophobic surface enhanced the intestinal cellular internalization of nanoparticles. Pharmacodynamic studies revealed that PLGA-*Hyd*-PEG nanoparticles significantly lowered blood glucose levels following intrajejunal administration in both normal and diabetic rats. In addition, hydrophobic components such as lipids, phospholipids [130], stearic acid, tocopherol acetate [131], and deoxycholic acid [69] have been utilized in the preparation or surface modification of nanoparticles to enhance their lipophilicity, thereby facilitating epithelial absorption. Consequently, hydrophobic modification of nanoparticles represents a promising strategy for overcoming the intestinal epithelial cell barrier to improve the oral absorption of PPs encapsulated within them.

##### 3.3.3.2 Positively charged surface modification of nanoparticles

The negatively charged intestinal epithelial cells not only facilitate the uptake of cationized PPs but also achieve a similar effect for nanoparticles modified with positive charges. Wang et al. [132] developed cationic liposomes (CLs) using a thin film hydration method with egg yolk lecithin (EPC), cholesterol, and the cationic lipid DOTAP as the carrier materials. Bovine serum albumin (BSA) was adsorbed onto the surface of the cationic liposomes, forming a protein corona that neutralized the charge and made the surface more hydrophilic to enhance the permeation of mucus. When PcCLs traversed the mucus layer, the BSA corona was shed, exposing the cationic liposomes with positive electrical properties. This exposed positive charge on the liposomes promoted enhanced uptake by the intestinal epithelial cells, facilitating better drug absorption. In vitro and *in vivo* studies demonstrated that the uptake and trans-epithelial permeability of PcCLs were 3 and 8 times greater than that of free insulin, respectively. Besides, other cationic substances like octa-arginine (R8) peptide [102], chitosan [133], and aminopropyl groups [134] have also been employed to modify nanoparticles, enabling oral insulin nanoparticles to overcome the intestinal epithelial barrier.

##### 3.3.3.3 Ligand-functionalized nanoparticles

Since the intestine is the primary site for nutrient absorption, it expresses a wide range of receptors and transporters. These include ASBT in the ileum, peptide transporter 1 in the jejunum, proton-coupled amino acid transporter 1 (PAT1) expressed along the small intestine, transferrin receptor (TfR) in duodenal crypt cells, folate receptors located in the duodenum and jejunum. Transporter or receptor-mediated endocytosis in IECs can efficiently internalize extracellular substances upon the binding of ligand molecules to receptors. This pathway is vital for the uptake of essential nutrients, including vitamins, transferrin, and hormones, which are critical for human health.

The modification of NPs with active targeting ligands enhances their uptake and transport, offering a promising strategy to improve the oral bioavailability of PPs through targeted interactions with receptors, transporters, and specialized cells within the intestinal epithelium [135]. Zou et al. [117] devised the acid-resistant metal-organic framework (MOF) nanoparticles (UiO-68-NH_2_) to encapsulate insulin robustly and UiO-68-NH_2_ NPs were further modified with transferrins on their surface to enhance oral insulin delivery efficiency (Fig. 4C). The transferrin-coated NPs exploited TfR-mediated transcellular pathways to achieve efficient translocation across the intestinal epithelium and controlled insulin release in physiological conditions, resulting in enhanced oral bioavailability and significant hypoglycemic effects. The area under the curve of the transferrin-insulin-UiO-68 complex was 120.7 mIU × hour/liter, achieving a relative bioavailability of 29.6 %. This value was 42.3 and 26.9 times greater than that observed for the oral insulin solution (0.7 %) and the insulin-UiO-68 (I@U) group (1.1 %), respectively.

These findings demonstrate the capability of MOF nanoparticles to shield proteins from the harsh gastric environment, and confirm that transferrin modification substantially enhances nanoparticle endocytosis by intestinal cells, thereby improving the oral bioavailability of insulin. In addition to transferrin, other nutritional components such as vitamins (VB_12_ [136], folate [137], and biotin [138]), saccharides (galactose [139], mannose), and transporter-associated fatty acids (DOCA [140] and butyrate), along with proteins similar to transferrin such as immunoglobulin G [141] and lectin [142], can target various receptors expressed on the surface of intestinal cells to facilitate the internalization of NPs, thereby improving the transcellular transport of orally administered PPs.

##### 3.3.3.4 Zwitterionic modification

Inspired by the rapid traversal of viral particles through the mucus layer and mucosa, viral-like zwitterionic surface modification has been extensively utilized in the surface engineering of oral PPs nanoparticles [143]. Nanoparticles modified with zwitterionic polymers exhibit a uniform surface charge distribution, resulting in an overall neutral charge. Moreover, the inherent hydrophilicity of zwitterionic polymers would mitigate mucin adsorption, thereby enhancing their permeation through the mucus layer [144]. Upon traversing the mucus layer, the apical surface of IECs, which are rich in PAT1 receptors, can effectively mediate the internalization of zwitterion-modified nanoparticles. Fang et al. [145] devised a novel approach for oral protein drug delivery by employing *in situ* polymerization of zwitterions to encapsulate proteins. These complexes, modified by polyzwitterions, demonstrate the capability to navigate through mucus and cellular barriers, presumably facilitated by the PAT1 mechanism. Remarkably, this technique achieved oral bioavailabilities of 16.9 % for insulin and 12.5 % for immunoglobin G. In particular, the administration of polyzwitterion-encapsulated insulin significantly reduced blood glucose levels in diabetic models across multiple species (mice, rats, and pigs).

Building on these promising results, zwitterionic coatings have also been integrated with MOF nanoparticles to further potentiate oral peptide delivery. Chen and co-workers designed a pH-triggered self-unpacking capsule system that encapsulates zwitterionic hydrogel-coated MOF nanoparticles (Ex@MIL101@Gel±) to enhance the oral delivery of Exendin-4 (Fig. 4D) [118]. This zwitterionic surface modification allowed the nanoparticles to evade mucus entrapment and significantly increased their intestinal absorption through PAT1-mediated cellular uptake. *In vivo* studies using a diabetic rat model showed that oral administration of these capsules resulted in a substantial and sustained increase in plasma Exendin-4 and insulin levels, leading to a pronounced hypoglycemic effect with a relative pharmacological availability of 17.26 %. In addition, other zwitterions like phosphatidylcholine, carboxybetaine, and sulfobetaine, phosphatidylcholine [146], polydopamine [147], poly(carboxybetaine) [148] and betaine [149] have also been used for the surface modification of nanoparticles to enhance their traversal across the mucus layer and uptake by intestinal cells. These research results underscore the significance of zwitterionic surface modification in enhancing the intestinal mucosal delivery of oral PPs nanoparticles.

### 3.4 Comparative analysis and integration of strategies

The various strategies for overcoming GI barriers in oral protein and peptide delivery demonstrate differing degrees of effectiveness and complexity. Each strategy targets specific barriers, addressing distinct challenges associated with biochemical barriers, mucus barriers, and the intestinal epithelial barrier. For a comprehensive analysis and detailed comparison of each strategy, including their advantages and disadvantages, refer to Table 1. While each strategy offers solutions to specific challenges encountered during the oral administration of PPs, they also have inherent limitations. For instance, while PEGylation effectively overcome biochemical barriers and promotes mucus penetration, it may impede the uptake of nanoparticles or PPs by IECs due to steric hindrance and electric inertness. Similarly, encapsulation provides robust protection against the harsh GI environment, but encapsulation alone could not significantly enhance cellular uptake without adequate design to target and penetrate the intestinal epithelium. Permeation enhancers can transiently open tight junctions between epithelial cells, improving paracellular transport of PPs. However, this approach can compromise the integrity of the intestinal barrier, potentially leading to undesirable side effects such as increased permeability to harmful substances and local irritation or inflammation. Enzyme inhibitors protect PPs from proteolytic degradation in the GI tract. Nonetheless, they may also interfere with the normal digestive processes and nutrient absorption, leading to potential GI disturbances. Chemical modifications, designed to improve stability and permeability of PPs, may introduce variability in drug activation and effectiveness.

**Table 1:** Individual strategies for overcoming GI barriers in oral protein/peptide delivery and comprehensive analysis.

Strategy	pH barrier	Enzyme barrier	Mucus barrier	Epithelial barrier	Advantages	Disadvantages	References
pH Modulation	✔ Adjusts local pH	✔ Inhibit enzymatic activity	✘	✘	Simple, cost-effective for stabilizing proteins in extreme pH environments	Limited to pH environments, not effective against enzymes or mucus	[31,32]
Chemical Modification	✔ Alters protein stability	✔ Prevents enzymatic degradation	✔ Reduces interaction with mucus	✔ Improves epithelial uptake	Increases stability, prevents degradation, and improves absorption	May alter biological activity; requires extensive validation	[34,38]
Encapsulation	✔ Protects from extreme pH	✔ Isolates from enzymes	✔ Reduces interaction with mucus	✔ Enhances epithelial uptake	Comprehensive protection and controlled release	Complex and costly to formulate, risk of incomplete release	[41]
Enzyme Inhibitors	✘	✔ Inhibits enzymes	✘	✘	Reduces enzymatic degradation, enhances protein stability	Potential toxicity, possible disruption of normal digestion	[44,45]
Mucolytic Agents	✘	✘	✔ Breaks down mucus for penetration	✘	Enhances penetration, improves bioavailability	May disrupt protective mucus; risk of infection	[70,71]
Hydrophilic/Slippery Modification	✘	✘	✔ Reduces interaction with mucus	✘	Increases mucus penetration and epithelial cell interaction	Requires specific design; effectiveness may vary	[67,74]
Surface Charge Adjustment	✘	✘	✔ Adjusts charge to penetrate mucus	✘	Optimizes penetration through mucus while balancing epithelial interaction	Requires precise control of charge	[81,82]
Size Optimization	✘	✘	✔ Allows easier passage through mucus pores	✘	Enhances penetration through the mucus mesh	Requires fine-tuning for optimal size; may complicate design	[85,86]
Shape Optimization	✘	✘	✔ Optimizes shape for better penetration	✘	Helical or rod-like shapes enhance movement through mucus	Requires specialized synthesis techniques	[88,89]
Rigidity Adjustment	✘	✘	✔ Allows deformation to penetrate mucus	✘	Flexible particles enhance passage through mucus without being trapped	Overly rigid particles may get trapped; overly soft ones may not penetrate efficiently	[93,94]
Micro-/Nanomotors	✘	✘	✔ Active propulsion through mucus	✔ Enhances epithelial uptake	Provides active transport for deeper penetration and better absorption	Complex and costly to manufacture; risk of controlling movement direction	[98,95]
Hydrophobic Modification	✘	✘	✔ Increases interaction with hydrophobic regions in mucus	✔ Enhances epithelial interaction	Enhances adhesion to mucus, improving residence time	May lead to clearance before absorption due to excessive interaction with the outer mucus layer	[103]
Positively Charged Modification	✘	✘	✔ Increases adhesion to negatively charged mucus	✔ Enhances epithelial interaction	Improves adhesion and absorption	May result in increased mucus retention and hinder passage through the epithelial barrier	[99,96]
Thiolation	✘	✘	✔ Forms disulfide bonds with mucus.	✘	Stronger adhesion to mucus for prolonged retention and protection.	Over-adhesion may limit transport through mucus.	[97]
Permeation Enhancers	✘	✘	✘	✔ Enhances epithelial absorption.	Temporarily opens tight junctions to allow protein/peptide passage through epithelial cells.	Potential for toxicity and disruption of normal epithelial function.	[110,111]
Ligand Modification	✘	✘	✘	✔ Enhances specific uptake by epithelial cells.	Enables targeted delivery to epithelial cells, improves absorption efficiency.	Requires specific targeting ligands and precise design.	[116,128,117]
Zwitterionic Modification	✘	✘	✔ Mimics viral-like surface to penetrate mucus.	✔ Enhances epithelial absorption.	Uniform charge distribution improves mucus penetration and absorption.	Complex to synthesize; may require costly materials.	[144,118]
Dissociable Coating	✘	✘	✔ Coating dissolves after mucus penetration.	✔ Exposes interactive surface for epithelial uptake.	Ensures penetration through mucus while enabling epithelial absorption.	Complex to design and optimize; requires precise control.	[75,150]
Hydrolyzable Coating	✘	✘	✔ Coating changes in specific response.	✔ Enhances epithelial uptake.	Improves mucus penetration and epithelial interaction via controlled coating degradation.	May require specific conditions for optimal activation.	[55,151]
Microneedles	✔Encapsulation protection	✔Encapsulation protection	✔ Directly pierces through the mucus layer.	✔ Directly delivers across the epithelial layer.	Bypasses traditional barriers; directly delivers peptides/proteins across the epithelial barrier.	Limited applicability to oral delivery systems; complex manufacturing process.	[152,153]

Given these limitations, it is clear that no single strategy can address all the GI barriers effectively. Since nanoparticles can serve as a platform to effectively integrate multiple strategies, they have emerged as a promising vehicle for overcoming the multifaceted barriers presented by the GI tract in the oral delivery of PPs. Nanoparticles could provide a protective barrier that shields the loaded PPs from the acidic and enzymatic conditions of the GI tract. However, due to the inherent characteristics of mucus and epithelial cells, the surface properties of nanoparticles required to overcome the mucus barrier and the intestinal epithelial cell barrier are completely opposite. For nanoparticles to traverse the mucus layer without being ensnared, their surfaces should be hydrophilic, electrically neutral, or possess a slight negative charge to mitigate adhesion by mucins. However, this surface configuration, while advantageous for bypassing mucus entrapment, inadvertently diminishes interaction of NPs with the IECs. Reduced interaction leads to the reduced absorption of nanoparticles by IECs. To address this conundrum, many strategies have been proposed, including dissociable coating, hydrolyzable coating and some novel microneedles.

#### 3.4.1 Dissociable coating

To enhance the oral bioavailability of PPs through nanoparticle delivery systems, it is essential to promote the uptake of nanoparticles by IECs. The primary strategy focuses on increasing the binding rate between nanoparticles and IECs, thereby boosting the probability of uptake. However, IECs are more likely to bind nanoparticles with hydrophobic and positively charged surfaces, which in turn inhibits the nanoparticles' efficient traversal through the mucus layer. For this reason, Gao and co-workers developed zwitterion sulfobetaine 12 (SB12)-functionalized MSNs that exhibited enhanced mucus penetration capabilities for oral drug delivery (Fig. 2E) [69]. The SB12 coating on the MSNs provided a hydrophilic and neutral surface, facilitating the nanoparticles' transit through the GI mucus layer. As the nanoparticles penetrated deeper into the mucus, the SB12 coating gradually detached, revealing the underlying deoxycholic acid (DOCA) modification. This exposure of the hydrophobic and cationic DOCA surface promoted the nanoparticles' uptake by IECs via apical sodium-dependent bile acid transporter (ASBT)-mediated endocytosis, thereby improving the bioavailability of oral insulin.

**Fig. 5:**
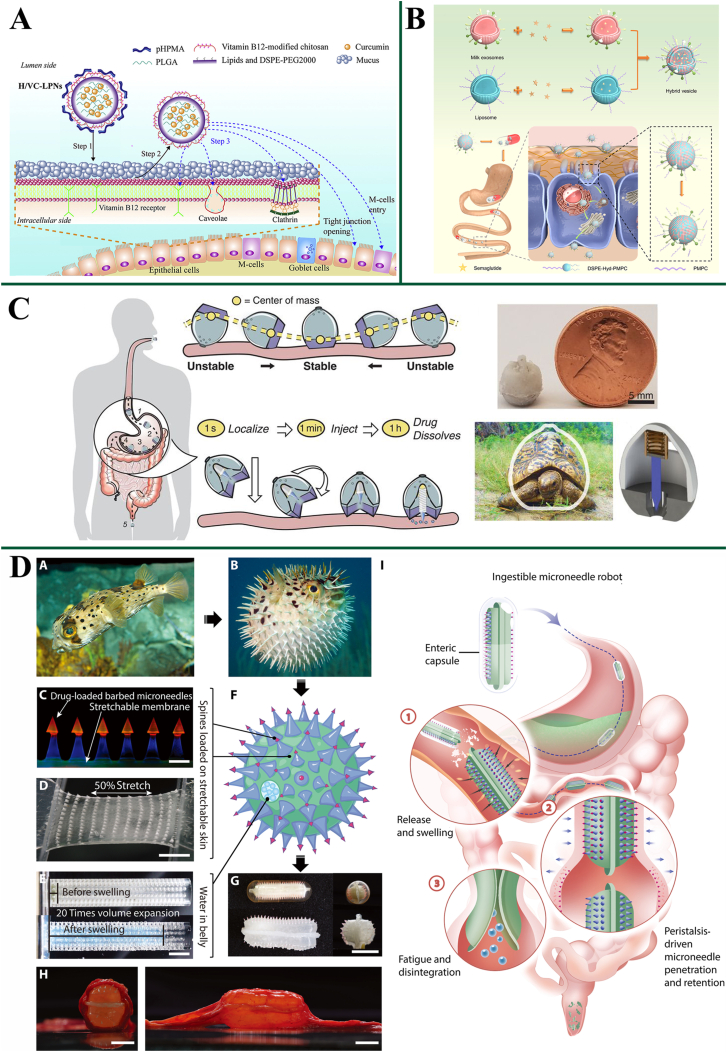
(A) The hydrophilic copolymer pHPMA associated with vitamin B12-modified chitosan-covered lipid polymeric nanoparticles (H/VC-LPNs) releases the pHPMA during mucus penetration [75]. Copyright 2019 Elsevier. (B) The mExos@DSPE-Hyd-PMPC vesicles exploit the differential pH microenvironment between the intestinal mucus layer and the epithelial cell surface to achieve pH-responsive hydrolyzable coating [55]. Copyright 2024 American Chemical Society. (C) Localization and injection for oral gastric delivery of insulin by mechanical SOMA [152]. Copyright 2019 Science. (D) Intestinal peristalsis–actuated porcupinefish-inspired microneedle robots for oral delivery of biologic drugs [153]. Copyright 2024 Science.

Dissociable coatings effectively address both the mucus layer and IECs barriers by enabling timely surface property switching of the nanoparticles when they encounter these barriers. Ding et al. [150] developed protein corona–coated cationic liposomes for the oral delivery of the GLP-1 analog liraglutide. Positively charged AT-1002–modified liposomes (AT-CLs) were cloaked with a hydrophilic BSA corona to form near-neutral, mucus-inert Pc-AT-CLs. The BSA coating markedly reduced mucoadhesion and aggregation, allowing the nanoparticles to diffuse more freely through the intestinal mucus. As the particles migrated across the mucus layer, the loosely adsorbed BSA corona progressively detached, exposing the cationic AT-CL cores at the epithelial surface, where they could interact more efficiently with intestinal epithelial cells. This adaptive core–shell architecture thus reconciles the contrasting surface properties required for efficient mucus penetration and subsequent epithelial uptake in oral PPs delivery. Liu et al. [75] also used pHPMA as a detachable coating to enhance the penetration of NPs (Fig. 5A). And the timely departure of pHPMA exposed ligand modified NPs to IECs resulting in improved uptake of nanoparticles. The dissociable coating effectively solves the problem of the opposite surface properties of nanoparticles required for crossing the mucus layer and being taken up by IECs.

#### 3.4.2 Hydrolyzable coating

Although electrically neutral nanoparticles rapidly crossed the mucus layer, it was observed that IECs exhibited poor binding with these electrically neutral nanoparticles [154]. Thus, the challenge arises in how to transform electrically neutral nanoparticles into the positively charged ones that would increase uptake of NPs by IECs. Developing pH-sensitive or enzyme-responsive systems may provide a viable solution to this issue, due to the differences in pH and enzyme activity between the mucus layer and the surface of IECs. Xiao and colleagues developed an innovative milk exosome-liposome hybrid vesicle system, mExos@DSPE-Hyd-PMPC, that exploits the differential pH environment between the intestinal mucus layer and the epithelial cell surface to achieve pH-responsive surface modification (Fig. 5B) [55]. Within the intestinal lumen, the mucus layer maintains a near-neutral pH, whereas the surface of IECs is characterized by a mildly acidic microenvironment due to localized proton release from the apical membrane, which is restricted by the mucus layer. This pH gradient induces the cleavage of pH-sensitive hydrazone bonds in the hybrid vesicles as they approach the epithelial surface, leading to a shift in surface charge from neutral to positive. This adaptive surface change is crucial for enhancing the vesicles' ability to penetrate the mucus layer and subsequently improve their uptake by epithelial cells. This self-adaptive, pH-sensitive system demonstrated a substantial enhancement in the oral bioavailability of semaglutide, achieving an oral bioavailability of 8.7 %.

In addition to pH-sensitive systems, enzyme-responsive charge-reversal strategies also offer an innovative solution to the challenge of overcoming both the mucus and epithelial barriers. These systems are designed to exploit the enzymatic microenvironment of the gastrointestinal tract, enabling nanoparticles to reversibly switch their surface charge in response to specific enzymatic triggers. This dynamic transformation allows the particles to maintain a neutral or negative charge for efficient mucus penetration and subsequently acquire a positive charge to promote epithelial uptake. Wu et al. [151] devised a mucus-penetrating virus-inspired biomimetic nanoparticle with charge-reversal ability, known as P-R8-Pho nanoparticles. These nanoparticles are densely coated with PLGA and feature a cationic R8 peptide along with specific anionic Pho, resulting in a small size and a virus-like neutral charged surface. This P-R8-Pho NPs achieve rapid mucus penetration and, through intestinal outer membrane-bound enzyme alkaline phosphatase-induced hydrolysis of surface-anchored Pho, leads to a timely charge-reversal that exposes the cationic R8 peptide. Consequently, this cationic R8 peptide triggers efficient cellular uptake of NPs by IECs *in vivo*. Oral administration of insulin-loaded P-R8-Pho nanoparticles induces a preferable hypoglycemic effect, achieving a 1.9-fold higher oral bioavailability compared with single CPP-modified nanoparticles in diabetic rats. This innovative approach combines biomimetic mucus-penetrating strategies with enzyme-responsive charge-reversal in a single nanovehicle, showcasing potential for overcoming the dual barriers of mucus and epithelial cells for effective oral delivery of PPs. The intestinal outer membrane-bound alkaline phosphatase constitutes a classic medium for facilitating the overturn of charge of nanoparticles following their traversal of the mucus layer [155,156]. The hydrolysis of surface coatings on nanoparticles has effectively converted their surface properties. These charge-reversible nanoparticles could effectively solve the problem of oral PPs nanoparticles overcoming the sequential barriers of the mucus layer and IECs simultaneously, which require nanoparticles to have opposite surface properties.

#### 3.4.3 Microneedles

Microneedle technology, traditionally utilized for transdermal drug delivery, effectively overcomes the stratum corneum barrier. By adjusting microneedle length, it enables enhanced drug penetration without damaging tissues or nerves, thus offering a pain-free administration approach [157]. Recent advancements have extended the application of microneedles beyond dermal systems to other mucosal delivery routes, including ocular, oral, and vaginal routes [158]. Notably, microneedle systems provide effective encapsulation and protection for loaded PPs, while also enabling precise puncturing of the mucus layer and intestinal epithelial barrier at the appropriate location, thereby achieving efficient oral delivery of PPs.

To translate these conceptual advantages into practical oral dosage forms, microneedle-based devices have been engineered for direct deployment within the GI tract. Firstly, Traverso et al. [159] designed a kind of metal microneedles. The microneedles can effectively penetrate GI mucus and epithelia, enhancing the oral bioavailability of biologically active macromolecules in swine models. Despite its safety and tolerability in trials, the biocompatibility of metallic microneedles remains a concern for clinical application. Consequently, biodegradable or dissolvable microneedles have been developed to mitigate metal toxicity risks. Abramson et al. [152] created a self-orienting millimeter-scale applicator (SOMA) system designed to enhance the bioavailability of orally administered insulin by mechanically inserting drug-loaded microneedles into the gastric mucosa (Fig. 5C). The SOMA autonomously orients itself in the stomach, ensuring proper alignment for microneedle insertion. The microneedles penetrated up to 7 mm into the mucosa without causing tissue damage and achieved near zero-order release kinetics, resulting in effective glycemic control. Building on this innovative gastric microneedle approach, Abramson's team presented another luminal unfolding microneedle injector (LUMI), a capsule device equipped with three arms bearing microneedle patches [160]. Upon reaching the small intestine, LUMI unfolds and delivers microneedles directly into intestinal tissue. Preclinical studies in swine demonstrated that the LUMI device could effectively deliver insulin with a systemic bioavailability exceeding 10 % compared to subcutaneous injection. Rani Therapeutics (San Jose, CA, USA) is advancing this type of microneedle technology for oral delivery of biologics, which has undergone clinical evaluation [161].

In addition to capsule- and unfolding-based microneedle platforms, more precise bioinspired devices have been developed to further enhance targeting precision and insertion efficiency. Gao et al. [153] designed a bioinspired intestinal microneedle robot that mimics the defense mechanism of porcupinefish (Fig. 5D). Upon reaching the intestine, the robot rapidly swells into a spiny structure by absorbing intestinal fluids, enabling its drug-loaded microneedles to be inserted into the mucosal tissue under the force of natural peristalsis. To protect the device from gastric degradation, it is encapsulated in an enteric-coated capsule until reaching the target site. *In vivo* experiments in minipigs demonstrated effective glucose reduction following duodenal delivery of insulin-loaded microneedle robots, with a reported insulin bioavailability of 23.6 %, significantly outperforming traditional intestinal gavage (0.6 %). Importantly, no signs of mucosal injury, bleeding, or perforation were observed, supporting the system's safety for GI drug delivery.

In parallel with these peristalsis-driven bioinspired microneedle robots, externally actuated microneedle systems have also been explored to achieve spatiotemporally controlled insertion within the intestine. Zhang et al. [162] developed magnetically controllable dip-printed independent microneedle motors (IMNMs) encapsulated in enteric capsules for oral insulin delivery, which successfully penetrated the intestinal mucosa under magnetic guidance and achieved sustained glucose-lowering effects in diabetic rabbits. Although microneedle-based oral delivery systems show promising results in improving bioavailability and controlling glucose levels, the long-term safety remains a concern. Repeated puncturing of the mucosal layer may cause chronic tissue irritation or inflammation, and the presence of foreign materials could trigger immune responses or foreign body reactions. Utilizing finer needles or biocompatible materials in the design of biodegradable microneedles may offer a promising strategy to enhance the safety and biocompatibility of oral microneedle systems for long-term administration.

#### 3.4.4 Lessons from failed or limited synergistic strategies

While numerous studies have demonstrated the success of synergistic strategies in overcoming multiple gastrointestinal barriers, several multi-strategy formulations have failed in clinical development. Analyzing these unsuccessful examples is critical for understanding the intrinsic limitations of multi-component delivery systems and refining future design approaches. A representative example is the oral insulin program developed by Nobex Corporation. Their formulation employed a dual modification strategy, in which insulin was covalently linked with a hydrophilic PEG chain to protect against enzymatic degradation, together with a lipophilic alkyl chain to enhance membrane permeation. To further augment absorption, the permeation enhancer sodium caprate (C10) was co-administered. Although this combinatorial design showed promising preclinical performance, the Phase III clinical trial failed to achieve the primary efficacy endpoint. Mechanistic analyses suggested that steric hindrance from PEGylation may have impaired the interaction of insulin with epithelial membranes, while high local concentrations of C10 caused cytotoxicity in intestinal epithelial models, collectively limiting the translation of this strategy to the clinic.

Another instructive example is Oramed's ORMD-0801, an oral insulin formulation that integrates multiple protective and absorption-enhancing approaches, including enteric coating, protease inhibitors, and permeation enhancers. Despite advancing to Phase III trials, the formulation exhibited an extremely low oral bioavailability (approximately 1 %) and produced glycemic effects insufficient to substitute for subcutaneous insulin therapy. The disappointing outcomes highlight the challenge of balancing enzymatic protection, permeation enhancement, and safety within a single dosage form. This case underscores that simply stacking multiple strategies does not guarantee synergistic improvements, and that rational optimization of each component and their interactions is critical to achieve clinically meaningful outcomes.

## 4 Marketed and clinical trials of oral PPs

The ultimate goal of developing various strategies to overcome the GI barriers during oral administration of PPs is to ensure their successful clinical application. Several representative oral PPs currently available on the market are listed in Table 2. Novo Nordisk's GLP-1A, Semaglutide (Rybelsus®), is an oral tablet that incorporates the permeation enhancer SNAC to enhance the bioavailability of Semaglutide [163]. In contrast, semaglutide is also marketed as injectable formulations, specifically Wegovy® and Ozempic®. Though these injectable forms are administered once weekly, a considerable number of patients prefer the oral formulation Rybelsus®, as oral administration offers superior convenience and improves adherence in the long-term management of chronic conditions such as diabetes and obesity. However, it should also be acknowledged that the bioavailability of Rybelsus® remains markedly low compared to injectable formulations, underscoring the need for further optimization of oral semaglutide delivery systems [164].

**Table 2:** Marketed and clinical trials of representative oral PPs formulations (Data obtained from https://clinicaltrials.gov/.).

Protein/Peptide	Conditions	Company	Product name	Strategy	Clinical status	ClinicalTrials.gov identifier
Semaglutide	Diabetes	Novo Nordisk A/S	Rybelsus®	Permeation enhancer: Sodium N-[8-(2-hydroxybenzoyl) Amino] Caprylate (SNAC)	Marketed	–
Taltirelin hydrate	Spinocerebellar degeneration	Mitsubishi Tanabe Pharma Corporation	Ceredist®	Chemical modification	Marketed	–
Desmopressin acetate hydrate	Central diabetes, insipidus, primary nocturnal enuresis	Ferring Pharmaceuticals	DDAVP®	Chemical modification	Marketed	–
Octreotide	Acromegaly	Chiasma	Mycapssa®	Cyclic peptide, enteric coating, permeation enhancer	Marketed	–
Cyclosporine A	Immunosuppression	Novartis AG	Neoral®/ Sandimmune®	Self-emulsifying Drug Delivery Systems (SNEDDS)	Marketed	–
Insulin	Diabetes	Oramed, Ltd.	ORMD-0801	Enteric coating, protease inhibitors and permeation enhancer	Phase 3	NCT02094534
		Biocon Limited	IN-105 (Insulin Tregopil)	Chemical modification	Phase 2-3	NCT03430856
		Novo Nordisk A/S	Insulin 338	Chemical modification, permeation enhancer (sodium caprate)	Phase 2	NCT02470039
		Diasome Pharmaceuticals	Oral hepatic directed vesicles (HDV)-Insulin	Liposomes containing insulin conjugated with hepatocyte-targeting moieties	Phase 2-3	NCT00814294
		Oshadi Drug Administration	Oshadi Icp	Insulin, proinsulin and C-peptide suspended in oil are loaded in hydrophobic polysaccharide modified silica nanoparticles	Phase 2	NCT01973920
Salmon Calcitonin	Postmenopausal osteoporosis	Tarsa Therapeutics, Inc.	Oral Calcitonin Tablets	Enteric coating, local pH modulator: citric acid protects sCT against peptidases by lowering down local pH	Phase 3	NCT00959764
		Novartis	SMC021	Permeation enhancer (5-CNAC)	Phase 2	NCT00421590
Cyclosporine A	Mild to Moderate Ulcerative Colitis	Sigmoid Pharma	CyCol™	Controlled release minicapsule formulation	Phase 2	NCT01033305
Leuprolide	Endometriosis	Enteris BioPharma Inc.	Ovarest®	Permeation enhancer, pH modulator and enzyme inhibitor	Phase 2	NCT02807363
IL-10	Ulcerative Colitis	Applied Molecular Transport	AMT-101	Fusion protein	Phase 2	NCT05372939
Dolcanatide	Colorectal Carcinoma	Ironwood Pharmaceuticals	Dolcanatide	Chemical modification: D-asparagine and D-leucine replace natural amino acids and carboxyl groups	Phase 1	NCT03300570
Parathyroid hormone	Hypoparathyroidism	RANI Therapeutics	Oral RaniPill® capsule	Microneedles	Phase 1	NCT05164614
		Entera Bio Ltd.	EB612 (EBP05)	Enteric coating and permeation enhancer	Phase 2	NCT03516773

Another example is Desmopressin acetate (DDAVP®), developed by Ferring Pharmaceuticals, which is a cyclic peptide. Its stability is enhanced through chemical modifications, including deamination of the first amino acid and substitution of the eighth amino acid, L-arginine, with D-arginine. While these chemical modifications improve the stability of DDAVP®, the lack of additional strategies results in an oral bioavailability of only approximately 0.1 % [4]. Chiasma's Octreotide, a synthetic analog of the endogenous hormone somatostatin, demonstrates higher stability in simulated gastric fluid (SGF) due to its cyclic structure. By incorporating enteric coating and permeation enhancers, its oral bioavailability is increased to 0.7 % [165]. Cyclosporine A, one of the most successful oral peptide therapeutics on the market, benefits from the combination of a cyclic lipophilic undecapeptide with Self-Emulsifying Drug Delivery Systems (SNEDDS), achieving oral bioavailability of 19–40 % [166].

In addition to marketed products, a number of oral PPs formulations are currently undergoing clinical trials (Table 2), employing strategies such as chemical modifications, peptide cyclization, enteric coating, enzyme inhibitors, local pH modulators, and permeation enhancers. Furthermore, nanotechnology is gradually transitioning from the laboratory to clinical applications. For example, Diasome Pharmaceuticals has developed an oral insulin liposome formulation that has completed Phase II clinical trials and is preparing for Phase III trials. Insulin is encapsulated within liposomes modified with hepatocyte-targeting moieties (biotin-phosphatidyl-ethanolamine). These hepatocyte-targeting liposomes are transported across the intestinal epithelium into the portal vein, subsequently captured by hepatocytes, thereby mimicking the physiological insulin delivery process [167]. Oshadi Drug Administration Ltd. (Israel) has pioneered the use of inorganic silica nanoparticles for oral insulin delivery, incorporating these nanoparticles (1–100 nm) to load insulin in an oily suspension along with branched polysaccharides [168]. It is particularly noteworthy that microneedle-based strategies for oral PPs delivery are gaining increasing recognition. Rani Therapeutics (USA) has developed a robotic pill for oral protein delivery, which consists of a sugar microneedle-based balloon structure encapsulated by PLGA, a degradable polymer. Insulin-loaded microneedles have shown promising hypoglycemic effects [169].

In summary, it is evident that most oral PPs, whether already approved for market use or undergoing clinical trials, primarily rely on straightforward strategies such as chemical modification, encapsulation, and permeation enhancers. These approaches are not only easy to implement but also enable efficient scale-up production. Moreover, their simplicity makes them more accessible for regulatory approval. Permeation enhancers have become key components in the oral PPs preparations. Numerous companies continue to develop a variety of permeation enhancers. For example, Emisphere has developed a series of caprylic acid derivatives, such as SNAC and 5-CNAC, to enhance permeability of oral PPs. However, research on more complex nanotechnology should not be overlooked, as oral PPs formulations based on liposomes and silica nanoparticles have already been approved for clinical trials. Furthermore, innovation should not be limited to traditional approaches. As long as strategies ensure the effective protection of PPs within the GI tract and facilitate their transport across the intestinal epithelial barrier, successful oral administration of PPs can be achieved. Notably, the innovative microneedles have enhanced the bioavailability of oral insulin to 50 %, a significant improvement compared to other oral PPs delivery systems. In conclusion, the continued development and clinical application of innovative strategies for oral PPs delivery hold great promise for improving their bioavailability and transforming the landscape of oral PPs.

## 5 Challenges of current strategies

Despite the rapid development of innovative approaches to enhance the oral delivery of PPs, most currently marketed oral formulations employ relatively simple strategies. These primarily include chemical modifications to improve the enzymatic stability of PPs, or co-administration with permeation enhancers to increase transmembrane absorption. However, these single-barrier-targeting strategies typically fail to address the complexity of the GI environment comprehensively. As a result, their oral bioavailability remains extremely low, often below 1 %, which significantly increases dosing requirements and production costs, ultimately limiting their broader clinical adoption [9]. In contrast, various advanced delivery systems based on nanotechnology have demonstrated promising capabilities in overcoming multiple physiological barriers and markedly improving oral bioavailability in preclinical studies.

Nevertheless, translating these systems into clinical practice faces considerable challenges. One critical issue lies in their scalability; achieving consistent nanoparticle size distribution and reproducible physicochemical properties during large-scale production remains difficult. Furthermore, the high cost of nanoparticle manufacturing poses an economic hurdle, particularly when the clinical benefit does not outweigh the expense. However, while the development costs of advanced delivery systems may initially be high, their long-term cost-effectiveness should be considered. For instance, oral formulations that improve patient adherence could lead to better disease management, fewer complications, and ultimately lower healthcare costs, despite the higher upfront costs. In addition, scaling up production processes and optimizing manufacturing efficiencies may reduce the per-dose cost over time, improving the overall cost-effectiveness of these technologies.

Regulatory acceptance is another substantial limitation. Innovative nanocarriers often lack sufficient long-term safety data from rigorous preclinical and clinical studies, making regulatory bodies cautious about their approval. Concerns regarding potential immunogenicity, accumulation, and off-target effects necessitate extensive toxicological profiling, which remains incomplete for most experimental systems. To bridge this gap, the use of biocompatible materials, such as FDA-approved polymers and phospholipids for micelles and liposomes, has been explored. These carriers offer enhanced safety profiles and facilitate a smoother translational path. However, these organic carriers frequently suffer from inherent instability, leading to premature drug leakage, crystallization, and reduced therapeutic efficacy [170].

Recently, inorganic carriers such as mesoporous silica nanoparticles (MSNs) and metal-organic frameworks (MOFs) have gained attention due to their excellent structural stability and high drug loading capacity. These systems effectively prevent premature drug release and maintain therapeutic activity. However, concerns over their long-term biodegradability and potential *in vivo* accumulation have limited their progression toward clinical application. For instance, degradation of MOF is influenced by factors such as metal ion and organic ligand nature, which can lead to the release of toxic species and subsequent organ toxicity [171]. Similarly, poor biodegradability of MSN may result in their accumulation in organs like the liver and kidneys, causing potential toxicity [172].

In contrast, organic carriers like liposomes and PLGA nanoparticles generally exhibit better biocompatibility and biodegradability, with lower *in vivo* accumulation and toxicity [173]. The emergence of degradable inorganic and hybrid carriers, such as biodegradable silica and polydopamine-based nanoparticles, provides a promising solution to this dilemma [174,175]. These systems offer the dual advantages of robust structural integrity and improved biosafety through controlled degradation. However, their long-term pharmacokinetics, systemic toxicity, and large-scale reproducibility still require comprehensive investigation before clinical translation can be realized. While significant advances have been made in overcoming individual GI barriers, the clinical translation of oral PPs remains hindered by low bioavailability, manufacturing complexity, regulatory uncertainty, and safety concerns. Future research should prioritize the development of multifunctional yet clinically feasible delivery platforms that balance efficacy, safety, scalability, and cost-effectiveness.

## 6 Conclusions and future perspectives

Due to the multiple formidable barriers of the GI tract, oral administration of PPs is still one of the most challenging problems in the pharmaceutical industry to date. A range of innovative technologies have been developed to enhance the oral bioavailability of PPs by addressing challenges related to their stability and permeability. This review has discussed recent progress in various strategies to overcome different GI barriers, ranging from chemical modifications, encapsulation techniques, and permeation enhancers to more advanced systems such as nanoparticles and microneedles. Several technologies have already been successfully applied in commercially available oral PPs formulations, including enteric coatings, enzyme inhibitors, permeation enhancers, and chemical modifications. Although these technologies have been used in clinically marketed oral PPs formulations, the bioavailability remains significantly low because they address only a subset of the challenges associated with GI oral PPs delivery and fail to effectively overcome all the barriers. Nanotechnology-based strategies have shown potential in the development of oral protein and peptide formulations. However, the clinical application of these novel approaches is still at an early stage, and several critical issues remain to be addressed before successful clinical translation can be achieved.

(1) Advanced biomodels for safety evaluation: Nanoparticle-based oral protein delivery systems pose potential safety risks, especially during long-term use. Before clinical translation, it is essential to conduct systematic and comprehensive toxicity evaluations. Emerging organoid models, derived from human stem cells, offer a more accurate representation of human tissue compared to traditional animal models. Future research should focus on optimizing organoid cultures to better replicate human GI physiology and integrating them with microfluidic devices to simulate realistic dynamic conditions, such as shear stress and pH fluctuations. Additionally, efforts should be directed toward enhancing the scalability of organoid models for high-throughput screening, enabling rapid safety and toxicity assessments of nanoparticle-based delivery systems. Incorporating organoid assays with computational models can further facilitate predictive modeling for long-term safety, addressing the need for more reliable preclinical testing. Finally, comparative studies between organoid-based models and conventional animal testing are necessary to validate their predictive power for human-relevant safety data.
(2) Nanoparticle engineering and scale-up: Translating laboratory formulations to industrial-scale production is a major challenge. While many nanotechnology-based platforms have demonstrated impressive results in preclinical studies, their successful translation into clinically viable products is often hindered by complex manufacturing processes, high production costs, and scalability limitations. Future research should focus on simplifying nanoparticle production techniques, such as investigating microfluidic and continuous-flow methods, to enhance scalability and maintain batch-to-batch consistency. Additionally, surface modification strategies, like PEGylation and ligand targeting, should be developed to improve nanoparticle bioavailability and efficiency without adding significant complexity to the manufacturing process. Efforts should also be directed toward cost-effective encapsulation technologies, optimizing materials such as biodegradable nanoparticles and liposomes to reduce production costs while ensuring stability during GI transit and effective targeting of epithelial cells. Furthermore, process optimization is crucial, with a focus on reducing material consumption, shortening processing times, and minimizing production steps, all while maintaining therapeutic efficacy, to ensure the scalability and commercial viability of nanoparticle-based delivery systems.
(3) Artificial intelligence (AI)–assisted development: AI is rapidly emerging as a powerful tool with the potential to empower various industries. Future research should focus on integrating AI into the development of oral PPs delivery systems, as it is crucial for accelerating progress and driving significant advancements in this field. AI can be utilized to mine large-scale toxicology datasets from preclinical studies and clinical trials, identifying key structural and physicochemical properties of delivery systems that correlate with biocompatibility and low immunogenicity. Machine learning models can be developed to predict nanoparticle behavior, such as tissue penetration, bioavailability, and toxicity, based on their chemical structure, size, and surface properties. By integrating these models with experimental data, researchers can create robust prediction frameworks to identify potentially toxic or ineffective formulations early, reducing reliance on costly animal studies. Moreover, AI can support preclinical decision-making by identifying promising formulations for further testing, optimizing formulations based on predicted outcomes, and minimizing time spent on ineffective candidates, ultimately streamlining the drug development process.

In conclusion, despite the significant challenges associated with orally administered PPs, advances in technology and multidisciplinary collaboration have led to the development of numerous innovative approaches. This review has comprehensively examined the current strategies designed to overcome various GI barriers, offering valuable insights and references for future research on oral PPs delivery. With ongoing technological progress, these efforts are expected to pave the way for the successful development of effective oral PPs delivery systems, ultimately improving therapeutic outcomes and patient compliance.

## CRediT authorship contribution statement

**Xiaofan Wang:** Writing – review & editing, Writing – original draft, Investigation, Conceptualization. **Keke Wang:** Writing – review & editing, Writing – original draft, Resources. **Yitan Fang:** Writing – review & editing, Writing – original draft, Software. **Youxi Zhang:** Writing – review & editing, Writing – original draft. **Lixin Yi:** Writing – review & editing, Resources. **Xiaohong Li:** Writing – review & editing, Validation, Investigation, Conceptualization. **Qinfu Zhao:** Writing – review & editing, Writing – original draft, Supervision, Conceptualization. **Xu Zhu:** Writing – review & editing, Writing – original draft, Conceptualization. **Shuang Cai:** Writing – review & editing, Writing – original draft, Conceptualization. **Long Wan:** Writing – review & editing, Writing – original draft, Supervision, Conceptualization.

## Declaration of generative AI and AI-assisted technologies in the writing process

During the preparation of this work the authors used ChatGPT /4.0o in order to improve language and readability. After using this ChatGPT/4.0o, the authors reviewed and edited the content as needed and take full responsibility for the content of the publication.

## Declaration of competing interest

The authors declare that they have no known competing financial interests or personal relationships that could have appeared to influence the work reported in this paper.

## Data Availability

No data was used for the research described in the article.
